# Loss of full-length hnRNP R isoform impairs DNA damage response in motoneurons by inhibiting Yb1 recruitment to chromatin

**DOI:** 10.1093/nar/gkab1120

**Published:** 2021-11-25

**Authors:** Hanaa Ghanawi, Luisa Hennlein, Abdolhossein Zare, Jakob Bader, Saeede Salehi, Daniel Hornburg, Changhe Ji, Rajeeve Sivadasan, Carsten Drepper, Felix Meissner, Matthias Mann, Sibylle Jablonka, Michael Briese, Michael Sendtner

**Affiliations:** Institute of Clinical Neurobiology, University Hospital Wuerzburg, Wuerzburg 97080, Germany; Institute of Clinical Neurobiology, University Hospital Wuerzburg, Wuerzburg 97080, Germany; Institute of Clinical Neurobiology, University Hospital Wuerzburg, Wuerzburg 97080, Germany; Department of Proteomics and Signal Transduction, Max Planck Institute of Biochemistry, Martinsried82152, Germany; Institute of Clinical Neurobiology, University Hospital Wuerzburg, Wuerzburg 97080, Germany; Experimental Systems Immunology, Max Planck Institute of Biochemistry, Martinsried 82152, Germany; Institute of Clinical Neurobiology, University Hospital Wuerzburg, Wuerzburg 97080, Germany; Institute of Clinical Neurobiology, University Hospital Wuerzburg, Wuerzburg 97080, Germany; Institute of Clinical Neurobiology, University Hospital Wuerzburg, Wuerzburg 97080, Germany; Department of Proteomics and Signal Transduction, Max Planck Institute of Biochemistry, Martinsried82152, Germany; Experimental Systems Immunology, Max Planck Institute of Biochemistry, Martinsried 82152, Germany; Department of Proteomics and Signal Transduction, Max Planck Institute of Biochemistry, Martinsried82152, Germany; NNF Center for Protein Research, Faculty of Health Sciences, University of Copenhagen, Copenhagen DK-2200, Denmark; Institute of Clinical Neurobiology, University Hospital Wuerzburg, Wuerzburg 97080, Germany; Institute of Clinical Neurobiology, University Hospital Wuerzburg, Wuerzburg 97080, Germany; Institute of Clinical Neurobiology, University Hospital Wuerzburg, Wuerzburg 97080, Germany

## Abstract

Neurons critically rely on the functions of RNA-binding proteins to maintain their polarity and resistance to neurotoxic stress. HnRNP R has a diverse range of post-transcriptional regulatory functions and is important for neuronal development by regulating axon growth. *Hnrnpr* pre-mRNA undergoes alternative splicing giving rise to a full-length protein and a shorter isoform lacking its N-terminal acidic domain. To investigate functions selectively associated with the full-length hnRNP R isoform, we generated a *Hnrnpr* knockout mouse (*Hnrnpr^tm1a/tm1a^*) in which expression of full-length hnRNP R was abolished while production of the truncated hnRNP R isoform was retained. Motoneurons cultured from *Hnrnpr^tm1a/tm1a^* mice did not show any axonal growth defects but exhibited enhanced accumulation of double-strand breaks and an impaired DNA damage response upon exposure to genotoxic agents. Proteomic analysis of the hnRNP R interactome revealed the multifunctional protein Yb1 as a top interactor. Yb1-depleted motoneurons were defective in DNA damage repair. We show that Yb1 is recruited to chromatin upon DNA damage where it interacts with γ-H2AX, a mechanism that is dependent on full-length hnRNP R. Our findings thus suggest a novel role of hnRNP R in maintaining genomic integrity and highlight the function of its N-terminal acidic domain in this context.

## INTRODUCTION

Neurons are highly polarized cells that utilize post-transcriptional processing mechanisms to establish and maintain their distinct functionality and morphological complexity. Thus, they critically depend on the activity of RNA-binding proteins (RBPs) for their function and maintenance. RBPs regulate post-transcriptional RNA processing at all stages including splicing, polyadenylation, subcellular transport, stabilization and translation (1,2). The heterogeneous ribonucleoprotein R (hnRNP R) is an abundant RBP in the nervous system and has been implicated in motoneuron development. In embryonic spinal motoneurons, hnRNP R is localized in the nucleus but is also present in the cytoplasm including axons and growth cones (3). Knockdown of hnRNP R in cultured embryonic motoneurons leads to reduced axon growth while cell survival is unaffected (4). More recently, the RNA interactome of hnRNP R in motoneurons has been identified by individual nucleotide resolution cross-linking and immunoprecipitation (iCLIP). In the nuclear compartment, hnRNP R binding sites were prevalent in introns of pre-mRNAs while in the cytoplasm hnRNP R binding was enriched in 3′ UTRs (5). This result indicates that hnRNP R is multifunctional and exerts distinct roles in the nucleus and cytoplasm. In axons, hnRNP R regulates the subcellular localization of *β-actin* mRNA, a defect of which might contribute to the axon growth defect observed by hnRNP R depletion in motoneurons (6). Interestingly, the top RNA interactor of hnRNP R is the non-coding RNA 7SK (5). It has been suggested that hnRNP R/7SK complexes are not only involved in transcriptional regulation in the nucleus but also regulate axonal mRNA transport, possibly as components of messenger ribonucleoprotein (mRNP) particles.

Several lines of evidence have implicated hnRNP R in pathophysiological mechanisms underlying motoneuron disorders. HnRNP R interacts with the Smn protein, a deficiency of which causes the motoneuron disease spinal muscular atrophy (SMA) (3,7–9). In mouse motoneurons, hnRNP R co-localizes with Smn in presynaptic axon terminals indicating that both proteins transport mRNAs in this compartment (7). Smn itself does not have any known RNA binding domain. However, hnRNP R requires Smn for the axonal transport of *β-actin* mRNA (6). In agreement with this finding, loss of Smn phenocopies the knockdown of hnRNP R in primary motoneurons including reduced transport of *β-actin* mRNA accompanied by defects in axon growth (6). In addition to Smn, hnRNP R has also been found to interact with several proteins associated with frontotemporal lobar degeneration (FTLD) and amyotrophic lateral sclerosis (ALS) such as TDP-43, FUS, MATR3, EWSR1 and TAF15 (10–12). In patients with FTLD-FUS, hnRNP R is detectable in neuronal cytoplasmic and intranuclear inclusions suggesting that hnRNP R dysfunction might contribute to the underlying disease pathogenesis (13). Recently, four individuals were reported with missense or truncating variants in the C-terminal domain of hnRNP R. These individuals presented developmental defects including brain abnormalities (14). Taken together, these observations imply a role of hnRNP R in nervous system development but also its maintenance during aging.

While transcriptome-wide studies have revealed that many RBPs are alternatively spliced (15), the functions of individual isoforms, particularly their roles in the nervous system, have remained mostly unclear. Here we report that alternative splicing of exon 2 of the *Hnrnpr* pre-mRNA gives rise to two hnRNP R protein isoforms: a long one of full-length (hnRNP R-FL) and a short one lacking the N-terminal acidic domain (hnRNP R-ΔN). To specifically dissect the functions of the full-length hnRNP R isoform, we generated a *Hnrnpr* knockout mouse model in which expression of full-length hnRNP R was abrogated while production of hnRNP R-ΔN was retained and upregulated by a compensatory mechanism. We observed that primary embryonic motoneurons cultured from this mouse model developed normally but showed defects in DNA damage repair following exposure to ionizing irradiation and etoposide. We observed that hnRNP R normally localizes to chromatin and binds to phosphorylated histone H2AX, a major protein involved in DNA damage response (DDR) signaling. Through proteomic analysis we identified Y-box binding protein 1 (Yb1), a multifunctional DNA/RNA-binding protein, as hnRNP R interaction partner. Similar to loss of full-length hnRNP R, depletion of Yb1 reduced DNA repair in motoneurons. Recruitment of Yb1 to chromatin in response to DNA damage was impaired in the absence of the full-length hnRNP R isoform. Taken together, using an isoform-specific knockout mouse we reveal a role for the acidic domain of hnRNP R in DNA damage repair.

## MATERIALS AND METHODS

### Animals and ethics statement

C57Bl/6 and *Hnrnpr* knockout mice were kept at the animal facilities of the Institute of Clinical Neurobiology at the University Hospital Wuerzburg. Mice were maintained under controlled conditions at 20–22°C on a 12 h/12 h light/dark cycle at 55–65% humidity and provided with *ad libitum* food and water supply. Each experiment was performed strictly following the regulations on animal protection of the German federal law and of the Association for Assessment and Accreditation of Laboratory Animal Care, in agreement with and under control of the local veterinary authority.

### Generation of *Hnrnpr* knockout mice

Embryonic stem cells (ESCs) harboring an insertion of a gene trap cassette in the *Hnrnpr* locus (EUCOMM clone EPD0609_5_ A12) were obtained from the European Mouse Mutant Cell Repository (EuMMCR, Munich). The knockout strategy designated ‘targeted mutation 1a’ (tm1a) or ‘knockout-first’ is based on the insertion of a gene-trap cassette containing the mouse *En2* splice acceptor and a SV40 polyadenylation signal (16). Correct insertion site of the gene-trap cassette was verified by Long Range PCR (Supplemental Figure S1A). The *Hnrnpr* gene-trap mouse was generated in the transgenics facility of B.S.R.C. ‘Alexander Fleming’, Greece, as part of the INFRAFRONTIER-I3 initiative. ESCs were injected into blastocysts of host C57Bl/6 mice and transferred into pseudopregnant C57Bl/6 surrogate mothers, and the resulting chimeras were subsequently bred to C57BL/6 mice. F1 offspring were genotyped by PCR using the following primers: HF1 (5′- TCCACATTCTGACTGCAGCA -3′) located in the 5′ homology arm, LAR3 (5′-CACAACGGGTTCTTCTGTTAGTCC-3′) located in the targeting cassette and HR1 (5′- GATAGCCCAATAGCACCCCC -3′) located in the 3′ homology arm (Supplemental Figure S1A). Mice were backcrossed for at least four generations onto C57Bl/6 background.

### Cell culture

Murine embryonic spinal motoneurons were isolated and enriched as previously described (17). Lumbar spinal cords were isolated from E12.5 embryos and cleaned by removing meninges and dorsal root ganglia. Spinal cords were then digested with 0.1% trypsin and gently triturated to obtain individual cells. Motoneurons were enriched by panning using a p75^NTR^ antibody (clone MLR2, Biosensis). Panning plates were prepared by coating 10 cm Nunclon™ surface dishes (Thermo Fisher Scientific) with p75^NTR^ antibody diluted in Tris buffer (10 mM Tris–HCl pH 9.5). Cells were plated on p75^NTR^-coated plates for 1 h to allow the p75^NTR^ receptor of motoneurons to attach to the antibody. Cells were counted and plated on coverslips or culture dishes coated with poly-dl-ornithine hydrobromide (PORN) (P8638, Sigma) and laminin-111 (23017-015, Thermo Fisher Scientific). Motoneurons were cultured in Neurobasal medium (NB) (Gibco) supplied with 2% B27 (Gibco), 2% heat-inactivated horse serum (Linaris), 500 μM GlutaMAX (Gibco) and the neurotrophic factor BDNF (5 ng/ml). Cells were kept at 37°C, 5% CO_2_, and the medium was changed on day 1 and then every second day.

NSC-34 cells (Cedarlane) and HEK293TN cells (System Biosciences) were cultured at 37°C and 5% CO_2_ in high glucose Dulbecco's modified Eagle medium (DMEM) (Gibco) supplemented with 10% fetal calf serum (Linaris), 2 mM GlutaMAX (Gibco) and 1% Penicillin–Streptomycin (Gibco). Cells were passaged when they were 80–90% confluent.

AML12 cells were cultured at 37°C and 5% CO_2_ in DMEM:F12 Medium (Gibco) supplemented with 10% fetal bovine serum, Insulin–Transferrin–Selenium (ITS -G) (Thermo Fisher) and 40 ng/ml dexamethasone (Sigma).

### Gene expression analysis by quantitative PCR

RNA was isolated from motoneurons, NSC-34 cells, and murine tissues with the NucleoSpin^®^ RNA kit (Macherey-Nagel). RNA was treated with DNase I to ensure the removal of genomic DNA. 0.5–1 μg of total RNA was used for reverse transcription using the RevertAid First Strand cDNA Synthesis Kit (Thermo Fisher Scientific). To ensure genomic DNA removal, a negative control reaction was set up in parallel in which the reverse transcriptase was omitted. The cDNA was diluted 1:5 in water and used for quantitative PCR (qPCR) using the Luminaris HiGreen qPCR Master Mix (Thermo Fisher Scientific) on a LightCycler 1.5 thermal cycler (Roche). Primers were designed using Primer3Plus (Version: 2.4.2) (18). Primer specificity was verified by melting curve analysis and gel electrophoresis of qPCR products.

The following primers were used: *Hnrnp*r-*FL*: 5′-CCAGCTCTGCCCTGCAGC-3′ (Forward), 5′-AAGATCCACATAAGCTACCAATCCTGTCT-3′ (Reverse); *Hnrnpr-FL chimeric*: 5′-TGAAATATTTCAGACAGGTCC-3′ (Forward), 5′-CCACATAACTACCAATCCTGTTGG-3′ (Reverse); *Hnrnpr-ΔN*: 5′-TGAAATATTTCAGACAGGTCC-3′ (Forward), 5′-CCACATAACTACCAATCCTGTTGG-3′ (Reverse); *Hnrnpr-ΔN Chimeric*: 5′-CAGCTCTGCCCTGGTCCC-3′ (Forward), 5′-CCACATAAGCTACCAATCCTGTTGG-3′ (Reverse); *Gapdh*: 5′-GCAAATTCAACGGCACA-3′ (Forward), 5′-GTCGTGGAGTCTACTGGTG-3′ (Reverse); Intron1: 5′-TGTCATAGGCGCTCCAGTTT-3′ (Forward), 5′-GCTTTTAAAGGGCAAGGGGG-3′ (Reverse); *Hnrnpr*: 5′-GCAGTTTGCTGCCATTTGTA-3′ (Forward), 5′-GCCCCAGAATAAGGGAACTC-3′ (Reverse); 7SL: 5′-CCTGTAGTCCCAGCTACTCG-3′ (Forward), 5′-CTGCTCCGTTTCCGACCTGG-3′ (Reverse).

### Absolute quantification of *Hnrnpr* transcripts

To generate standard curves for each transcript, PCR amplicons were purified from agarose gels and cloned into pJET1.2 using the CloneJET PCR Cloning Kit (Thermo Fisher Scientific). The concentration of the plasmids was determined by photospectrometry, and their molecular weight was calculated. Serial dilutions of 10^1^ to 10^8^ plasmid copies were made and the Ct values for each dilution sample were measured. Ct values were plotted against the logarithmized plasmid copy number and a linear regression line was fitted. Absolute copy numbers of transcripts were calculated according to the linear regression line and presented as absolute copy number per 1000 copies of *Gapdh* or 7SL transcripts.

### 5′ Rapid amplification of cDNA ends (5′ RACE)

The 5′ RACE was carried out on total RNA extracted from brains of wildtype, heterozygous and homozygous *Hnrnpr^tm1a/tm1a^* mice. 1 μg of RNA was reverse-transcribed with the RevertAid First Strand cDNA Synthesis kit (Thermo Fischer Scientific) using oligo(dT)18 primers. The cDNA was diluted 1:5 in water, and subsequently purified using the NucleoSpin^®^ Gel and PCR Clean-up kit (Machery-Nagel). Poly-A tailing of the purified cDNA was performed in 50 μl reactions containing 1 μl Terminal Deoxynucleotidyl Transferase (Thermo Fisher Scientific), 5 μl of 10× terminal transferase buffer and 5 μl of 1 mM dATP. Reactions were incubated at 37°C for 30 min and then heated at 70°C for 10 min to stop the reaction. Amplification of the tailed cDNA was performed with gene-specific primer *Hnrnpr*-SP1, together with oligodT_adapter primer and adapter primer, using AccuPrime DNA Polymerase (Thermo Fisher Scientific). A nested PCR reaction was performed using primer *Hnrnpr*-SP2 located upstream of *Hnrnpr*-SP1 and the adapter primer. The PCR product was cloned into pJET1.2/blunt using CloneJET PCR Cloning Kit (Thermo Fisher Scientific) and sequenced. The following Primers were used: oligodT_adapter: 5′-GGCCACGCGTCGACTAGTACTTTTTTTTTTTTTTTTT-3′; adapter: 5′-CCACGCGTCGACTAGTACTTT-3′; *Hnrnpr*-SP1: 5′-GTACACACTGTCTGGTGGGG-3′; *Hnrnpr*-SP2: 5′-TCCCGTGGTCACATCCAAAG-3′.

### Western blot analysis

Murine tissues were lysed in modified RIPA Buffer (50 mM Tris–HCl pH 7.4, 150 mM NaCl, 0.5% sodium deoxycholate, cOmplete™ protease inhibitor cocktail (Roche)) using a handheld homogenizer. Total protein concentration was measured using the BCA protein assay kit (Thermo Fisher Scientific). Protein lysates were denatured in Laemmli Buffer (63 mM Tris–HCl pH 6.8, 2% sodium dodecyl sulfate (SDS), 0.1% β-mercaptoethanol, 10% glycerol and 0.0005% bromophenol blue) for 10 min at 99°C. Equal amounts of protein were subjected to SDS-PAGE and transferred to nitrocellulose membrane (GE Healthcare). Membranes were probed with indicated primary antibodies diluted in Tris-buffered saline with Tween 20 (TBST) (50 mM Tris–HCl pH 7.6, 150 mM NaCl, 1% Tween 20) with 5% milk overnight at 4°C. After three washes with TBST, corresponding peroxidase-conjugated secondary antibodies were added for 1 h at room temperature (RT). Blots were washed three times with TBST and treated with enhanced chemiluminescent reagent (Thermo Fisher Scientific) followed by exposure on X-ray film (Fuji super RX). Blots were scanned and quantified by densitometry analysis with Fiji (19). The following primary and secondary antibodies were used: rabbit polyclonal anti-hnRNP R (1:2000, ab30930, Abcam), mouse monoclonal anti-β-Actin (1:4000, GTX26276, GeneTex), goat polyclonal anti-Calnexin (1:20 000, AB0037-200, Sicgen), mouse monoclonal anti-Gapdh (1:10 000, CB1001, EMD Millipore), rabbit polyclonal anti-Histone H3 (1:10 000, ab1791, Abcam), mouse monoclonal anti-phospho-Histone H2A.X (Ser139) (1:10 000, clone JBW301, Merck), rat monoclonal anti-HA (1:10 000, clone 3F10, Roche), rabbit monoclonal anti-Yb1 (1:5000, ab76149, Abcam), goat anti-mouse IgG (1:10 000, 115-035-003, Jackson ImmunoResearch), donkey anti-rabbit IgG (1:10 000, 711-035-152, Jackson ImmunoResearch), mouse anti-rabbit IgG, light chain-specific (1:10 000, 211-032-171, Jackson ImmunoResearch), goat anti-rat IgG (1:10 000, 112-035-003, Jackson ImmunoResearch), donkey anti-goat IgG (1:10 000, 705-035-003, Jackson ImmunoResearch) and VeriBlot for IP Detection Reagent (HRP) (1:1000, ab131366, Abcam).

### Immunofluorescence staining and image acquisition

Motoneurons grown for 6 DIV on laminin-coated glass coverslips were washed twice with phosphate-buffered saline (PBS) (Thermo Fisher Scientific) to remove serum and debris, then fixed in 4% methanol-free formaldehyde (Thermo Fisher Scientific) for 15 min. Cells were then permeabilized with 0.3% Triton X-100 in PBS for 20 min. After three washes in PBS, blocking solution containing 15% goat or donkey serum, depending on the species of the secondary antibody, 2% BSA and 5% sucrose in PBS was applied onto coverslips and incubated at RT for 1 h. Cells were incubated overnight with primary antibodies diluted in blocking solution at 4°C. Cells were washed three times with PBS, incubated with appropriate fluorescently labeled secondary antibodies diluted in PBS for 1 h at RT. The following primary and secondary antibodies were used: rabbit polyclonal anti-hnRNP R (1:1000, ab30930, Abcam), rabbit polyclonal anti-hnRNP R (1:1000, HPA026092, Sigma), mouse monoclonal anti-phospho-Histone H2A.X (Ser139) (1:1000, clone JBW301, Merck), rabbit monoclonal anti-Yb1 (1:1000, ab76149, Abcam), mouse monoclonal β-Tubulin (1:1000, 1111876, Roche), goat anti-rabbit (H + L; Cy5, 1:500, A21070; Invitrogen), donkey anti-rabbit IgG (H + L; Cy3, 1:500, 711-165-152, Jackson ImmunoResearch), goat anti-mouse IgG (H + L; Cy3, 1:500, 115–165–146, Jackson ImmunoResearch), goat anti-mouse IgG1 (Alexa Fluor 488, 1:500, A21121, Thermo Fisher Scientific), goat anti-mouse IgG (H + L; Cy5, 1:500, 115–175–146; Jackson ImmunoResearch). Nuclei were counterstained with DAPI and coverslips were mounted on glass-slides with Aqua Poly/Mount (18606, Polysciences). 16-bit images with 800 × 800–pixel resolution were acquired with an Olympus Fluoview 1000 confocal system equipped with a 60× objective (oil, numerical aperture: 1.35). Images were processed with Fiji. Maximum intensity projections were created from 0.5 μm z-stacks. For intensity measurements, identical image acquisition and processing settings were applied, and mean intensities were obtained.

### High-resolution *in situ* hybridization

High-resolution fluorescence *in situ* hybridization (FISH) was carried out following the manufacturer's instructions (Panomics) with minor modifications. Motoneurons grown for 6 DIV were fixed with paraformaldehyde lysine phosphate (PLP) buffer (4% paraformaldehyde, 5% glucose, and 0.01 M sodium metaperiodate, pH 7.4) for 15 min at RT and washed with RNase-free PBS. Cells were permeabilized using the supplied detergent solution (Panomics) for 4 min at RT and incubated with ViewRNA ISH probe sets for murine 7SK diluted 1:100 in hybridization buffer for 3 h at 40°C. Following probe hybridization, cells were washed with PBS and then subjected to sequential hybridization with pre-amplifier DNA, amplifier DNA and label probe oligonucleotides diluted 1:25 in corresponding buffers for 1 h each at 40°C. Cells were then washed three times with the supplied wash buffer at RT. Following FISH, immunostaining was performed as described above.

### Knockdown via lentiviral delivery of shRNA

To specifically knockdown the full-length hnRNP R isoform, shRNA oligonucleotides targeting *Hnrnpr* exon 2 were designed and cloned into a modified version of pSIH-H1 shRNA vector (System Biosciences) expressing EGFP according to the manufacturer's instructions. Empty pSIH-H1 expressing EGFP was used as control. The following antisense sequences were used for shRNA cloning: shRNA-FL-A: 5′-ATCAAGTCTTTCTGCCACC-3′, shRNA-FL-B: 5′-TTCATCAAGTCTTTCTGCC-3′ and shRNA-FL-C: 5′-TACCGCATTACCATTCACC-3′. An shRNA targeting both hnRNP R isoforms was generated as previously described (7). To generate the rescue constructs, shRNA oligonucleotides targeting the 3′ UTR of *Hnrnpr* were designed using the following antisense sequence: 5′-ATTTAAATGAGTAGGAGGC-3′. The following antisense sequence was used for design of a shRNA targeting Yb1: 5′-CCTGTAACATTTGCTGCCTCCGC-3′. Lentiviral particles were packaged in HEK293TN cells with pCMV-VSVG and pCMVΔR8.91 helper plasmids as described previously (20). Cells were transfected with TransIT-293 (Mirus Bio) in Opti-MEM™ I Reduced Serum Medium (Thermo Scientific) with 10% fetal calf serum for 12–14 h, and viral supernatants were harvested 72 h after transfection by ultracentrifugation. For lentiviral knockdown experiments, motoneurons and NSC-34 cells were incubated with lentivirus for 10 min at RT before plating.

### Expression plasmids

HA-tagged hnRNP R isoforms or deletion mutants were overexpressed in HEK293TN cells as following. The coding sequences of hnRNP R-FL, hnRNP R-ΔN, hnRNP R-ΔRRM, hnRNP R-ΔRGG or EGFP fused to a HA tag at the C-terminus were PCR-amplified and inserted into pSIH-H1 digested with NheI and SalI using the NEBuilder^®^ HiFi DNA Assembly Cloning Kit (New England Biolabs, USA). HEK293TN cells were transfected with Lipofectamine 2000 Transfection Reagent (Thermo Fisher Scientific) in Opti-MEM™ I Reduced Serum Medium. Cells were harvested 24 h after transfection and processed for western blot analysis.

The plasmids for rescue of hnRNP R-depleted motoneurons were constructed by inserting sequences encoding EGFP-tagged hnRNP R-FL or hnRNP R-ΔN into pSIH-H1 containing a shRNA targeting the 3′ UTR of *Hnrnpr*. For EGFP-tagged hnRNP R-FL, the exonic and intronic portions of exon 1 to exon 3, the *Hnrnpr* coding sequence from exon 4 onwards and the coding sequence of EGFP were PCR-amplified and cloned into pSIH-H1 digested with NheI and SalI using the NEBuilder^®^ HiFi DNA Assembly Cloning Kit (New England Biolabs, USA). The translation initiation codon located in exon 4, which normally gives rise to hnRNP R-ΔN, was mutated from ATG to ACG, leading to exclusive expression of hnRNP R-FL from this construct. For EGFP-tagged hnRNP R-ΔN, sequences encoding hnRNP R-ΔN and EGFP were PCR amplified and inserted into pSIH-H1 containing the *Hnrnpr* shRNA.

### Subcellular fractionation of NSC-34 cells

Subcellular fractionation was performed as previously described (21) with the following modifications. NSC-34 cells were seeded on a 10 cm cell culture dish and grown to 80–90% confluency. Cells were washed once with ice-cold PBS and incubated in 4 ml cytosolic fractionation buffer (10 mM HEPES, 100 mM KCl, 5 mM MgCl_2_, 35 μg/ml digitonin) on ice for 10 min. The extract was collected and centrifuged at 2000 × g for 10 min at 4°C. Following centrifugation, the supernatant was collected as the cytosolic fraction (Cyt). The cell culture dish was washed once with ice-cold PBS and 4 ml nuclear fractionation buffer (10 mM HEPES, 100 mM KCl, 5 mM MgCl_2_, 0.5% NP-40) was added. The cell lysate was collected, incubated on ice for 15 min and centrifuged at 20 000 × g for 15 min at 4°C. After centrifugation, chromatin-bound proteins were precipitated in the pellet. The supernatant containing organellar and nuclear luminal proteins was collected as the nuclear soluble fraction (Nuc). The pellet containing the chromatin-bound fraction (Chr) was washed once with ice-cold PBS and dissolved in 4 ml RIPA buffer (50 mM Tris–HCl pH 8.0, 150 mM NaCl, 1% NP-40, 0.5% sodium deoxycholate, 0.1% SDS), followed by sonication at 100% amplitude, cycle 0.5, for 1 min on ice with an Ultrasonic Processor UP50H/UP100H (Hielscher Ultrasonics), and incubation with 1 U of Benzonase (Santa Cruz Biotechnology) at 4°C for 30 min on a rotator to allow complete digestion of nucleic acids.

To investigate the RNA- and DNA-dependency of chromatin-bound proteins, the protocol from (22) was used with modifications. After removal of the cytosolic fraction, cells were washed once with ice-cold PBS and lysed in nuclear fractionation buffer. The nuclear lysate was distributed equally to 1.5 ml tubes and incubated on ice for 15 min. Samples were centrifuged at 20 000 × g for 15 min at 4°C, and supernatants were discarded. The pellets were washed once with PBS and resuspended in equal amounts of RIPA buffer. One aliquot was sonicated, treated with 1 U of Benzonase and incubated on a rotator at 4°C for 30 min. For RNA digestion, samples were treated with RNase A (Thermo Fisher Scientific) for 20 min at RT. As control, RNase A was omitted.

### Immunoprecipitation

NSC-34 cells were grown on a 10 cm dish to 80–90% confluency. Cells were lysed in 1 ml of IP lysis buffer (50 mM Tris–HCl pH 7.4, 140 mM NaCl, 1% Triton X-100, cOmplete™ protease inhibitor cocktail) supplemented with 1 U of Benzonase on a rotator at 4°C for 1 h. 20 μl of protein A or G Dynabeads (Thermo Fisher Scientific) were washed three times with IP lysis buffer and incubated with 2 μg of antibody or IgG control in IP lysis buffer on a rotator at 4°C for 2 h. The following antibodies were used for immunoprecipitation: mouse monoclonal anti-Yb1 (sc-101198, Santa Cruz Biotechnology), mouse monoclonal anti-phospho-Histone H2A.X (Ser139) (clone JBW301, Merck) and normal mouse IgG (sc-2025, Santa Cruz Biotechnology). 400 μl of cell lysate was added to the antibody-bound beads and incubated on a rotator at 4°C overnight. Following incubation, beads were separated from lysate on a magnetic stand Beads were washed three times using IP buffer followed by addition of 50 μl of 1 × Laemmli buffer and heating at 99°C for 10 min. The eluate was separated from the magnetic beads and transferred to a new reaction tube for gel loading or stored at −20°C until use.

### RNA immunoprecipitation

NSC-34 cells were grown on 10 cm dish until 80–90% confluency. Cells were lysed in 1 ml of IP lysis buffer (50 mM Tris pH 7.4, 140 mM NaCl, 1% Triton X-100, cOmplete™ protease inhibitor cocktail) on a rotator for 20 min at 4°C. 20 μl of protein G Dynabeads (Thermo Fisher Scientific) were washed three times with IP lysis buffer and incubated with 2 μg of antibody or IgG control in IP lysis buffer on a rotator at 4°C for 2 h. The following antibodies were used for immunoprecipitation: mouse monoclonal anti-Yb1 (sc-101198, Santa Cruz Biotechnology), and normal mouse IgG (sc-2025, Santa Cruz Biotechnology). 400 μl of cell lysate was added to the antibody-bound beads and incubated on a rotator at 4°C for 1 h. Following incubation, beads were separated from lysate on a magnetic stand and 80 μl of IP lysis buffer was added to the beads and mixed by pipetting up and down. 30 μl of the mix were removed and used for protein extraction by adding 6 μl 5 × Laemmli buffer and heating at 99°C for 10 min. Co-precipitated RNAs were isolated by adding 350 μl buffer A1 (NucleoSpin^®^ RNA kit (Macherey-Nagel)) and 350 μl ethanol to the remaining beads followed by RNA extraction according to the manufacturer's instructions. Total RNA was used for reverse transcription using the RevertAid First Strand cDNA Synthesis Kit (Thermo Fisher Scientific). In addition to primers against *Hnrnpr-FL* and *Hnrnpr-ΔN*, the following primers were used: *Eef2:* 5′-TGTCAGTCATCGCCCATGTG-3′ (Forward), 5′-CATCCTTGCGAGTGTCAGTGA-3′ (Reverse).

### Single cell gel electrophoresis (comet assay)

The alkaline comet assay was performed on motoneurons under steady state conditions or after exposure to 9 Gy of γ-irradiation. The protocol from (23) was used with modifications. Comet slides were prepared the day before the procedure, by immersion of superfrosted glass microscope slides into 1% of regular melting agarose solution to form a uniform layer. 50 000 motoneurons/well were plated in a 48-well plate and grown for 6 DIV. Cultures were rinsed once with PBS, treated with 0.05% trypsin (Thermo Fisher Scientific) for 3 min and collected in 150 μl of NB medium supplemented with horse serum and B27 to neutralize the trypsin. Samples were mixed with 150 μl of 1% low melting-point agarose (LMA) (final 0.5%) and immediately layered on the pre-coated slides. The cell suspension-agarose mixture was allowed to solidify at 4°C before applying a second layer of LMA. The slides were dipped in pre-chilled lysis solution (1.2 M NaCl, 100 mM Na_2_EDTA, 0.26 M NaOH, pH > 13) for 24 h at 4°C. Slides were washed three times for 20 min each in electrophoresis solution (2 mM Na_2_EDTA, 0.03 M NaOH, pH 12.3) for DNA unwinding and conversion of alkali-labile sites to single strand breaks, then subjected to electrophoresis under constant current of 7 V for 25 min in the dark. The slides were then washed in deionized water for 5 min. Comet slides were fixed with pre-chilled methanol and allowed to air dry. DNA breaks were visualized by staining with propidium iodide (0.1 mg/ml) for 20 min, after which the slides were washed with water and coverslipped. Image acquisition was performed using the Olympus Fluoview 1000 confocal system with a 20× objective equipped with appropriate filters. Opencomet plugin from Fiji (24) was used to assess DNA damage by measuring comet tail moment.

### Immunoprecipitation of hnRNP R complexes for mass spectrometry (#1 IP-MS)

Primary mouse motoneurons were cultured for 7 DIV with a seeding density of 1 000 000 cells. The cultures were washed three times with PBS and cells were lysed with IP lysis buffer (50 mM Tris pH 7.4, 140 mM NaCl, 1% Triton X-100, and cOmplete™ protease inhibitor cocktail). Beads crosslinked to anti-hnRNP R antibody or control IgG were added to the lysates and incubated for 5 h at 4°C. Following immunoprecipitation, beads were divided into two fractions of equal volume. One fraction was washed three times with wash buffer without Benzonase and the other three times with Benzonase-containing wash buffer for 10 min each at RT. This was followed by one washing of both fractions with wash buffer without Benzonase. The washed beads were eluted with elution buffer containing 0.1 M glycine, pH 2.5. The eluted fraction was processed for mass spectrometry analysis.

### Immunoprecipitation of HA-tagged hnRNP R complexes from transfected HEK293TN cells for mass spectrometry (#2 IP-MS)

HEK293TN cells were grown on six-well dishes until 80–90% confluency and transfected with plasmids for expression of HA-tagged hnRNP R-FL, hnRNP R-ΔN or EGFP. Twenty-four hours after transfection, cells were exposed to γ-irradiation as indicated and lysed in 1 ml of IP lysis buffer (50 mM Tris pH 7.4, 140 mM NaCl, 1% Triton X-100, cOmplete™ protease inhibitor cocktail) in the absence or supplemented with 1 U of Benzonase on a rotator at 4°C for 1 h. 10 μl of Pierce™ Anti-HA Magnetic Beads (Thermo Fisher Scientific) were washed three times with IP lysis buffer and cell lysate was added to the beads and incubated on a rotator at 4°C overnight. Following incubation, beads were separated from lysate on a magnetic stand and washed four times using IP buffer without Triton X-100. Beads were snap-frozen in liquid N_2_ for mass spectrometry (MS) analysis. Experiments were performed in quadruplicates.

### LC–MS/MS

Precipitated proteins were resuspended in 8 M urea (20 mM HEPES pH 8), reduced with 10 mM DTT for 30 min followed by alkylation of cysteines with 55 mM iodoacetamide for 45 min. 1 μg of LysC was added for initial proteolysis of proteins for 3 h at RT. Urea was diluted by a factor of five with 50 mM ammonium bicarbonate and the mixture was digested overnight with 1 μg trypsin. Proteomic workflows for the first (#1 IP-MS) and second (#2 IP-MS) experiments differed slightly due to optimizations in instrumentation and sample preparation procedures. Peptides were desalted via C18 StageTips (#1 IP-MS) or via SDB-RPS (#2 IP-MS) (25). Liquid chromatography (Thermo Scientific EASY-nLC 1000 HPLC) with in-house packed columns (75 μm inner diameter, 30 cm (#1 IP-MS) or 50 cm (#2 IP-MS) length, 1.9 μm C18 particles by Dr Maisch GmbH, Germany) was used to separate peptides in a gradient increasing the ratio of buffer B (acetonitrile, 0.5% formic acid) over A (0.5% formic acid) in 180 min (#1 IP-MS) or 60 min (2# IP-MS). Quadrupole Orbitrap mass spectrometers (Q Exactive for #1 IP-MS and Exploris 480 for #2 IP-MS, both Thermo Fisher Scientific) were coupled to the HPLC system via a nano electrospray source. The mass spectrometers were operated in a data-dependent mode. The survey scan range was set to 300 to 1650 *m*/*z*, with a resolution of 70 000 (#1 IP-MS) or 60 000 (#2 IP-MS). Up to the 10 most abundant isotope patterns with a charge ≥2 were subjected to HCD fragmentation at normalized collision energy of 27 (#1 IP-MS) or 30 (#2 IP-MS), an isolation window of 3 Th (#1 IP-MS) or 1.4 Th (#2 IP-MS) and a resolution of 17 500 (#1 IP-MS) or 15 000 (#2 IP-MS). Data were acquired using the Xcalibur software (Thermo Fisher Scientific).

### Data analysis and statistics

The MaxQuant software (versions 1.2.2.0 and 1.2.3 for #1 IP-MS and 1.6.0.15 for #2 IP-MS) (26) and Andromeda search engine (27) were employed to process MS raw data searching against the mouse fasta database (ipi.MOUSE.v3.68.) using standard settings (28). Protein intensities were normalized with MaxLFQ (29) and filtered for common contaminants, decoys and proteins only identified by site. Protein intensities are provided as Supplemental Table S1 (#1 IP-MS) and Table S2 (#2 IP-MS). For data analysis and visualization, Perseus was used. MaxLFQ intensities were log_2_-transformed and missing values were imputed with a normal distribution (width = 0.3; shift = 1.8). In case of #2 IP-MS, proteins were filtered for data completeness before imputation. Only proteins with at least three out of four quantifications in at least one condition (construct, irradiation, Benzonase treatment-specific) were kept, other proteins were removed. Significantly enriched proteins in pairwise interactome comparisons were determined by t-test statistics applying a permutation-based (250 permutations) false discovery rate of 5% and S0 of 0.1 (30).

### Statistical analysis

Statistical analysis methods were performed using GraphPad Prism 4.0. Methods are detailed in the figure legends.

## RESULTS

### The *Hnrnpr* gene encodes two hnRNP R protein isoforms generated by alternative splicing

**Figure 1.**
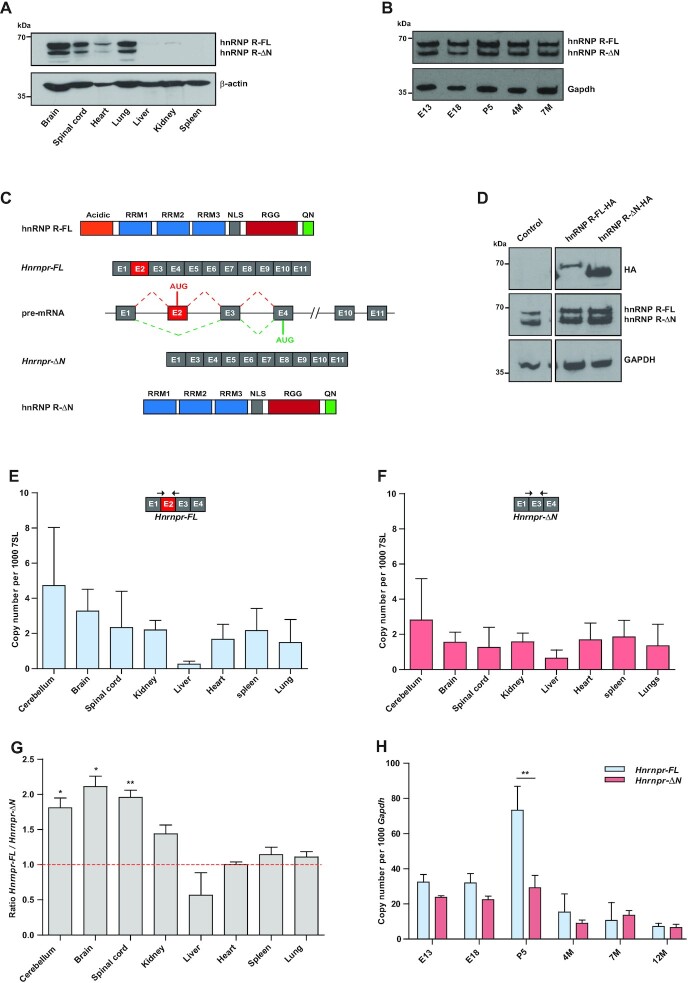
The *Hnrnpr* gene encodes two hnRNP R protein isoforms generated by alternative splicing. (**A**) Western blot analysis of hnRNP R isoforms using a C-terminal-specific antibody performed on tissue lysates from P5 wildtype mice. Equal protein loading in individual lanes was verified by probing with an antibody against β-Actin. (**B**) Western blot analysis of hnRNP R on brain lysates prepared from wildtype mice of different ages using the C-terminal-specific hnRNP R antibody. Equal protein loading in individual lanes was verified by probing with an antibody against Gapdh. (**C**) Schematic representation of the murine *Hnrnpr* locus that gives rise to a full-length *Hnrnpr-FL* mRNA and an exon 2-skipped *Hnrnpr-ΔN* mRNA produced by alternative splicing. Full‐length hnRNP R-FL and N‐terminally truncated hnRNP R-ΔN isoforms are shown. Protein domains are depicted as colored boxes: RRM (RNA-Recognition Motif), NLS (Nuclear Localization Signal), RGG (Arg-Gly-Gly box), and Q/N (Gln/Asn-rich domain). Constitutive exons are represented by gray boxes. Exon 2 (red box) is alternatively spliced. Introns are represented by horizontal lines. (**D**) Western blot analysis of lysates from HEK293TN cells transfected with plasmid constructs for expression of HA-tagged hnRNP R-FL and hnRNP R-ΔN. Total lysates were derived from HEK293TN cells 48 h after transfection with the indicated plasmid, and probed with anti-hnRNP R or anti-HA antibodies. GAPDH was probed as loading control. (**E**, **F**) Absolute copy numbers of *Hnrnpr-FL* (E) and *Hnrnpr-ΔN* (F) mRNA isoforms in tissues from P5 wildtype mice. Exon junction-spanning primers were used to measure isoform expression by qPCR. 7SL was used for normalization. Data are mean ± Standard Deviation (SD) (*n* = 3 animals). (**G**) Ratio of *Hnrnpr-FL* to *Hnrnpr-ΔN* isoform expression measured by qPCR. Data are mean ± SD (*n* = 3 animals). Statistical analysis was performed using a one-sample *t*-test comparing the ratios to a hypothetical mean of 1; *P ≤ 0.05, **P ≤ 0.01. (**H**) qPCR analysis of *Hnrnpr-FL* and *Hnrnpr-ΔN* isoform expression in brains of wildtype mice of different ages. The *Gapdh* transcript was used for normalization. Data are mean ± SD (*n* = 3 animals). Statistical analysis was performed using two-way ANOVA followed by Bonferroni post-hoc test; **P ≤ 0.01.

To characterize hnRNP R protein expression, we used an antibody against the C-terminus of hnRNP R for immunoblot analysis of different mouse tissues. The highest amounts of hnRNP R were detectable in the brain, spinal cord, heart and lung, while only low amounts were observed in the liver, kidney and spleen (Figure 1A). In all tissues examined, two hnRNP R protein isoforms were detectable. Immunoblot analysis of brain lysates from prenatal (embryonic day E13 and E18), newborn (postnatal day P5 and P12) and adult (4 months (4M) and 7 months (7M)) mice showed that both hnRNP R isoforms are expressed at all ages (Figure 1B). Visual inspection of the *Hnrnpr* locus in the UCSC genome browser revealed that the *Hnrnpr* pre-mRNA undergoes alternative splicing of exon 2 giving rise to two protein-coding mRNAs (Figure 1C). The full-length 632-amino acid protein (hnRNP R-FL), encoded by transcript variant 1 (Ref-Seq: NM_028871.2), is produced from an AUG translation initiation site located in exon 2 and contains a N-terminally located acidic domain, three consensus RNA-binding domains (RRM), a nuclear localization signal (NLS), a RGG domain, and C-terminal cluster of glutamine and asparagine residues (QN) (31). Upon exon 2 skipping (transcript variant 2, Ref-Seq: NM_001277123.1), a downstream AUG translation initiation site within exon 4 is utilized giving rise to a N-terminally truncated 531-amino acid protein (hnRNP R-ΔN).

It was originally thought that the N-terminally truncated isoform results from proteolytic cleavage of the full-length isoform (31). To address this possibility, constructs encoding either hnRNP R-FL (pSIH-hnRNP R-FL-HA) or hnRNP R-ΔN (pSIH-hnRNP R-ΔN-HA) fused to an HA tag were generated. Transfection of HEK293TN cells with pSIH-hnRNP R-FL-HA led to expression of a single product of around 70 kDa, as detected by an antibody against HA (Figure 1D). No other band was observed indicating that hnRNP R-FL does not undergo any proteolytic cleavage. Transfection with pSIH-hnRNP R-ΔN-HA produced a band of 60 kDa, corresponding to the truncated isoform (Figure 1D). These results suggest that the N-terminally truncated isoform is not a cleavage product of the full-length isoform in HEK293TN cells but rather produced by exon 2 skipping of *Hnrnpr* pre-mRNA.

To further investigate alternative splicing of *Hnrnpr*, we used exon-exon junction-spanning primers for specific detection of the *Hnrnpr-FL* (exon 2 included) and *Hnrnpr-ΔN* (exon 2 skipped) mRNA transcript isoforms by qPCR. Absolute quantification of both isoforms across tissues revealed that they are present at the highest levels in neuronal tissues, such as cerebellum, brain and spinal cord (Figure 1E, F). Quantification of the *Hnrnpr-FL*-to-*Hnrnpr-ΔN* isoform ratio showed that *Hnrnpr-FL* isoform is the predominant form in neuronal tissues and kidney, while both isoforms share equal expression in heart, spleen and lung, and the *Hnrnpr-ΔN* isoform predominates in the liver (Figure 1G). In brain, both the *Hnrnpr-FL* and *Hnrnpr-ΔN* transcripts were expressed at higher levels during development with peak expression at P5 (Figure 1H). At this age, the *Hnrnpr-FL* transcript was strongly increased compared to other ages whereas the *Hnrnpr-ΔN* isoform was only moderately upregulated (Figure 1H). These data suggest that hnRNP R-ΔN is a genuine isoform resulting from alternative splicing of *Hnrnpr* and that hnRNP R-FL is of particular importance in the nervous system during development.

### Generation and characterization of a mouse model for knockout of the full-length isoform of *Hnrnpr*

**Figure 2.**
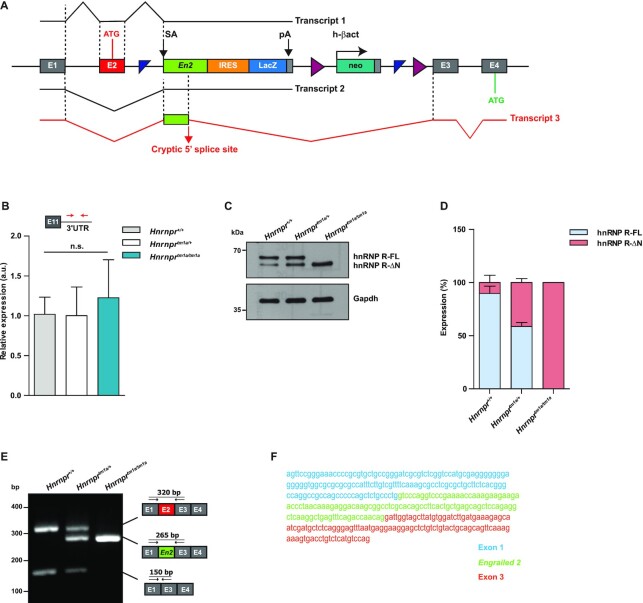
A mouse model for knockout of the full-length isoform of *Hnrnpr*. (**A**) Schematic representation of the targeting strategy for disruption of the *Hnrnpr* locus. A gene-trap cassette containing a splice acceptor (SA) from the engrailed-2 (*En2*) gene, an internal ribosome entry site (IRES) directing the translation of a reporter (LacZ) and a SV40 polyadenylation sequence (pA) was inserted upstream of *Hnrnpr* exon 3. In *Hnrnpr^tm1a/tm1a^* mice, splicing of exon 1 or exon 2 to *En2* generates transcripts that terminate at the SV40 pA site (Transcripts 1 and 2). Utilization of a cryptic 5′ splice site in *En2* results in a stable transcript (Transcript 3) with insertion of 115 nucleotides of *En2* between exons 2 and 3 of *Hnrnpr-ΔN* mRNA. (**B**) qPCR analysis of total *Hnrnpr* (*Hnrnpr-FL + Hnrnpr-ΔN*) levels using primers specific to the 3′ UTR in brains of P5 *Hnrnpr^+/+,^Hnrnpr^tm1a/+^* and *Hnrnpr^tm1a/tm1a^* mice. Data are mean ± SD (*n* = 4 animals). Statistical analysis was performed using one-way ANOVA followed by Tukey's multiple comparison test; n.s., not significant. (**C**) Western blot analysis of brain lysates obtained from P5 *Hnrnpr^+/+^*, *Hnrnpr^tm1a/+^* and *Hnrnpr^tm1a/tm1a^* mice using an antibody against the C-terminal domain of hnRNP R. Gapdh was used as loading control. (**D**) Quantification of western blot data in (C). The expression of each isoform is presented as the percentage of total. Data are mean ± SD (*n* = 4 animals). (**E**) RT-PCR analysis of RNA from brains of P5 *Hnrnpr^+/+^*, *Hnrnpr^tm1a/+^* and *Hnrnpr^tm1a/tm1a^* mice using primers within *Hnrnpr* exon 1 and 3. Schematic representations of observed amplicons are shown. (**F**) Sequence of the chimeric *Hnrnpr-ΔN-En2* splice product detected in (E) containing exon 1, 115 nucleotides of *En2* and *Hnrnpr* exon 3.

To investigate functions of hnRNP R-FL, we generated a mouse model that allowed us to selectively deplete its expression. This *Hnrnpr^tm1a/tm1a^* knockout mouse carries a gene trap cassette in the *Hnrnpr* locus upstream of exon 3 that contains a splice acceptor (SA) from the *engrailed-2* (*En2*) gene, an internal ribosome entry site (IRES) that directs the translation of β-galactosidase (LacZ), and a SV40 polyadenylation (pA) signal (16) (Figure 2A, Supplemental Figure S1A). Homozygous *Hnrnpr*^*tm1a/tm1a*^ mice were viable, and the three genotypes (*tm1a/tm1a*, *tm1a/+*, *+/+*) occurred according to the expected Mendelian ratio. *Hnrnpr*^*tm1a/tm1a*^ mice developed without overt phenotypic abnormalities or weight loss up to adulthood and remained healthy at least up to 16 months of age (Supplemental Figure S1B).

The gene trap cassette inserted upstream of exon 3 is expected to abrogate *Hnrnpr* transcription through splicing of exon 1 or exon 2 to the *En2* splice acceptor followed by termination of transcription through the SV40 pA signal (Figure 2A). However, when we determined *Hnrnpr* expression levels by qPCR using primers annealing within its 3′ UTR we found that *Hnrnpr* mRNA levels were unchanged in *Hnrnpr*^*tm1a/tm1a*^ mice (Figure 2B). Analysis of protein expression in brain lysates of P5 animals, using an antibody against the C-terminus of hnRNP R, revealed that the hnRNP R-FL isoform was absent, while the hnRNP R-ΔN isoform was still expressed in homozygous *Hnrnpr^tm1a/tm1a^* animals. Compared to heterozygous *Hnrnpr*^*tm1a/+*^ and wildtype *Hnrnpr^+^*^*/+*^ mice, levels of hnRNP R-ΔN were even increased in brains of homozygous *Hnrnpr*^*tm1a/tm1a*^ mice (Figure 2C,D). Absence of hnRNP R-FL isoform and expression of hnRNP R-ΔN was also detectable in heart and kidney of *Hnrnpr*^*tm1a/tm1a*^ mice, without any upregulation of hnRNP R-ΔN in these tissues compared to wildtype mice (Supplemental Figure S1C).

To determine the molecular basis underlying expression of the hnRNP R-ΔN isoform in *Hnrnpr*^*tm1a/tm1a*^ mice, we performed RT-PCR using primers located in exon 1 and exon 3. PCR products corresponding to the *Hnrnpr-FL* and *Hnrnpr-ΔN* isoform were detectable in wildtype and heterozygous mice (Figure 2E). An additional amplicon was obtained in heterozygous and homozygous animals. Sequencing of this amplicon revealed a cryptic donor splice site within the *En2* exon located 115 nucleotides downstream of the acceptor splice site. As a result, the *En2* exon was spliced to *Hnrnpr* exon 3, thereby skipping the SV40 pA signal (Figure 2A). When *Hnrnpr* exon 2 is skipped, this chimeric transcript containing the *En2* sequence would still give rise to the hnRNP R-ΔN isoform, which utilizes the translation initiation codon in exon 4. In contrast, for the *Hnrnpr-FL* isoform, inclusion of the *En2* exon is predicted to cause a frameshift in the coding sequence generating a premature termination codon in exon 3, which would render these transcripts prone to nonsense-mediated mRNA decay. We validated the production of this chimeric exon1-*En2*-exon3 aberrant splice product in *Hnrnpr^tm1a/tm1a^*and *Hnrnpr^tm1a/+^* by 5′ rapid amplification of cDNA ends (RACE) (Figure 2F). Taken together, our results indicate that expression of the hnRNP R-FL isoform is selectively abolished while expression of hnRNP R-ΔN is retained in *Hnrnpr^tm1a/tm1a^*mice giving rise to an isoform-specific *Hnrnpr* knockout mouse model.

### Depletion of full-length hnRNP R leads to upregulation of the hnRNP R-ΔN isoform

**Figure 3.**
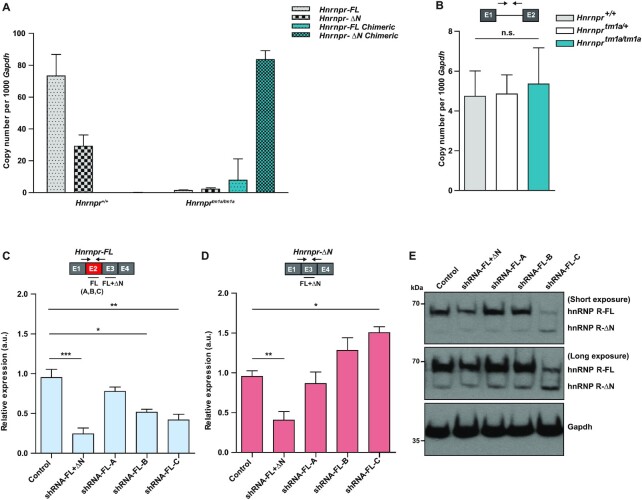
Depletion of full-length hnRNP R results in an up-regulation of the hnRNP R-ΔN isoform. (**A**) Absolute mRNA copy numbers of wildtype and chimeric *Hnrnpr* isoforms normalized to copies of *Gapdh* in brains of *Hnrnpr^+^^/+^*,*Hnrnpr^tm1a/+^* and *Hnrnpr^tm1a/tm1a^* mice. Data are mean ± SD (*n* = 4 animals). (**B**) qPCR analysis of *Hnrnpr* pre-mRNA levels using primers specific to intron 1 in brains of *Hnrnpr^+^^/+^*, *Hnrnpr^tm1a/+^* and *Hnrnpr^tm1a/tm1a^* mice. Data are mean ± SD (*n* = 4 animals). Statistical analysis was performed using one-way ANOVA followed by Tukey's multiple comparison test; n.s., not significant. (**C**, **D**) qPCR analysis of *Hnrnpr-FL* (C) or *Hnrnpr-ΔN* (D) levels in primary motoneurons transduced with an shRNA against exon 3 for depletion of both hnRNP R isoforms (shRNA-FL+ΔN), or with different shRNAs against exon 2 for depletion of the full-length hnRNP R isoform (shRNA-FL-A, shRNA-FL-B and shRNA-FL-C). Absolute copy numbers of *Hnrnpr-FL* (C) and *Hnrnpr-ΔN* (D) mRNA isoforms were normalized to absolute copies of *Gapdh*, controls were set to 1. Data are mean ± SD (*n* = 4 independent experiments). Statistical analysis was performed using one-way ANOVA followed by Tukey's multiple comparison test; *P ≤ 0.05, **P ≤ 0.01, ***P ≤ 0.001. (**E**) Western blot analysis of hnRNP R isoforms using a C-terminal-specific antibody performed on motoneurons transduced with control lentivirus or with lentiviruses for expression of the indicated shRNAs. Gapdh was used as loading control.

The increased expression of hnRNP R-ΔN in brains of *Hnrnpr^tm1a/tm1a^* mice compared to wildtype mice (Figure 2C, D) may reflect a compensatory response to the depletion of the full-length isoform. To investigate this further, we performed qPCR using junction-spanning primers and measured levels of the four possible splice variants by absolute quantification using standard curves (Supplemental Figure S2). In brains of wildtype mice, the copy numbers of the endogenous *Hnrnpr-FL* and *Hnrnpr-ΔN* isoforms were 73 ± 13 and 29 ± 7 copies, respectively, per 1000 copies of *Gapdh* (Figure 3A) in accordance with the immunoblot analysis (Figure 2C). In brains of *Hnrnpr^tm1a/tm1a^* animals, the endogenous *Hnrnpr-FL* and *Hnrnpr-ΔN* transcript isoforms were nearly absent and only the chimeric *En2*-containing transcripts were detectable. While we detected only low levels of the chimeric *Hnrnpr-FL* isoform, the chimeric *Hnrnpr-ΔN* mRNA isoform was much more abundant with a copy number of 83 ± 5 copies per 1000 copies of *Gapdh* (Figure 3A). Thus, compared to the *Hnrnpr-ΔN* isoform in wildtype mice, the chimeric *Hnrnpr-ΔN* mRNA was upregulated 2.8-fold in *Hnrnpr^tm1a/tm1a^* mice. These findings suggest that the increased levels of hnRNP R-ΔN protein in *Hnrnpr^tm1a/tm1a^* mice are the result of increased amounts of the corresponding chimeric *En2-*containing *Hnrnpr-ΔN* mRNA. In order to address whether the increase in hnRNP R-ΔN in *Hnrnpr^tm1a/tm1a^* mice was due to an elevation in transcription, we performed qPCR using primers annealing to intron 1 of *Hnrnpr* pre-mRNA transcripts. Our results revealed no difference in *Hnrnpr* pre-mRNA levels between *Hnrnpr^+/+^*, *Hnrnpr^tm1a/+^* and *Hnrnpr^tm1a/tm1a^* mice (Figure 3B) indicating that the observed upregulation of the hnRNP R-ΔN isoform in knockout animals is not due to an elevation in the transcription rate.

We next tested if the upregulation of *Hnrnpr-ΔN* mRNA isoform could be recapitulated by selective knockdown of the *Hnrnpr-FL* isoform in cultured primary mouse motoneurons. To this end, we designed three different short hairpin RNAs (shRNAs), designated shRNA-FL-A, shRNA-FL-B and shRNA-FL-C, targeting exon 2 of the *Hnrnpr-FL* isoform. As positive control, we used an shRNA (shRNA-FL+ΔN) targeting exon 3 to deplete both mRNA isoforms (7). Following lentiviral transduction with these shRNAs, motoneurons were cultured for 7 days *in vitro* (DIV) and levels of *Hnrnpr-FL* (Figure 3C) and *Hnrnpr-ΔN* (Figure 3D) mRNA isoforms were measured by qPCR. As expected, when using the shRNA targeting exon 3, the expression of both isoforms was reduced. In contrast, selective shRNA-mediated depletion of the full-length isoform led to upregulation of the *Hnrnpr-ΔN* isoform. This effect was dependent on the efficiency of the full-length isoform knockdown achieved by the shRNAs. The upregulation of the hnRNP R-ΔN isoform following knockdown of the full-length mRNA was also detectable at the protein level (Figure 3E). These data from cultured motoneurons thus recapitulate our observations made in brains of *Hnrnpr^tm1a/tm1a^* mice, further indicating that absence of hnRNP R-FL results in a compensatory post-transcriptional increase of the levels of the *Hnrnpr-ΔN* isoform in neurons.

### The hnRNP R-ΔN isoform is sufficient to support axon growth in primary motoneurons

**Figure 4.**
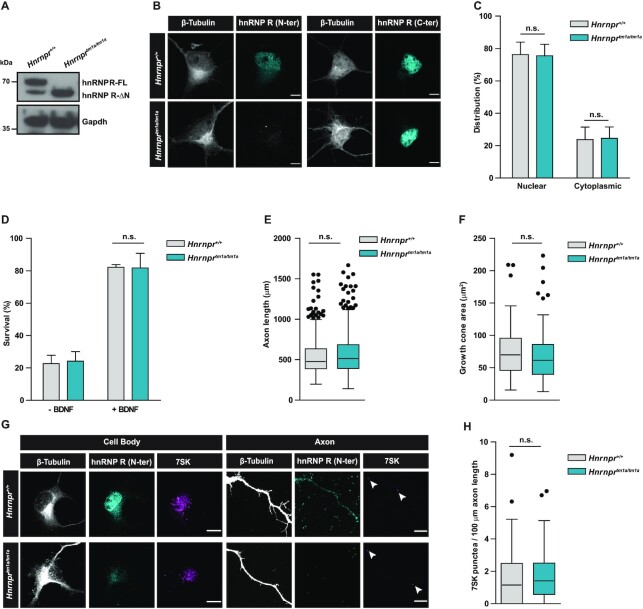
The hnRNP R-ΔN isoform is sufficient to support axon growth in primary motoneurons. (**A**) Western blot analysis of lysates from *Hnrnpr^+^^/+^* and *Hnrnpr^tm1a/tm1a^* motoneurons cultured for 7 DIV, probed with an antibody against the C-terminus of hnRNP R. Gapdh was used as loading control. (**B**) Representative images of primary motoneurons cultured for 7 DIV and immunostained with antibodies against the C-terminal or N-terminal domain of hnRNP R, and with an antibody against β-Tubulin. Scale bars: 5 μm. (**C**) Quantification of mean fluorescence intensity of the hnRNP R immunosignal obtained with the C-terminal-specific antibody in motoneurons shown in (B). Data are mean ± SD (*n* = 3 independent experiments; *N* = 30 motoneurons each for *Hnrnpr^+^^/+^* and *Hnrnpr^tm1a/tm1a^*). Statistical analysis was performed using two-way ANOVA followed by Bonferroni post-hoc test; n.s., not significant. (**D**) Quantification of the survival of motoneurons at 7 DIV as percentage of the number of motoneurons at 1 DIV, in the presence or absence of BDNF. Data are mean ± SD (*n* = 3 independent experiments; motoneurons were from *N* = 4 embryos each for *Hnrnpr^+^^/+^* and *Hnrnpr^tm1a/tm1a^*). Statistical analysis was performed using two-way ANOVA followed by Bonferroni post-hoc test; n.s., not significant. (**E**) Box-and-Whisker plots of the axon lengths of motoneurons cultured for 7 DIV. Data are mean ± SD (*n* = 3 independent experiments; *N* = 461 motoneurons for *Hnrnpr^+^^/+^* and *N* = 502 motoneurons for *Hnrnpr^tm1a/tm1a^*). Statistical analysis was performed using the Mann–Whitney test; n.s., not significant. (**F**) Box-and-Whisker plots of growth cone sizes of motoneurons cultured for 7 DIV. Data are mean ± SD (*n* = 3 independent experiments; *N* = 73 growth cones for *Hnrnpr^+^^/+^* and *N* = 86 growth cones for *Hnrnpr^tm1a/tm1a^*). Statistical analysis was performed using the Mann–Whitney test; n.s., not significant. (**G**) Representative images showing 7SK RNA labeling by *in situ* hybridization and immunostaining for hnRNP R in motoneurons derived from *Hnrnpr^+^^/+^* and *Hnrnpr^tm1a/tm1a^* mice. Arrowheads show 7SK-positive punctae. Scale bars: 5 μm. (**H**) Box-and-Whisker plots of the number of 7SK-positive punctae in axons of motoneurons cultured for 6 DIV. Data are mean ± SD (*n* = 3 independent experiments; *N* = 48 axons for *Hnrnpr^+^^/+^* and *N* = 55 axons for *Hnrnpr^tm1a/tm1a^*). Statistical analysis was performed using the Mann–Whitney test; n.s., not significant.

It has previously been shown that depletion of both hnRNP R isoforms in mouse primary motoneurons leads to defects in axon growth accompanied by reduced axonal transport of *β-actin* mRNA (4) and 7SK RNA (5). To dissect the role of hnRNP R-FL in these processes, we cultured motoneurons from *Hnrnpr^tm1a/tm1a^* mice and investigated axon growth and RNA localization. Similar to our observations in brain lysates (Figure 2C), immunoblot analysis of motoneurons derived from *Hnrnpr^tm1a/tm1a^* mice revealed loss of hnRNP R-FL and enhanced expression of hnRNP R-ΔN compared to motoneurons from *Hnrnpr^+/+^* mice (Figure 4A). Immunostaining with an antibody against the N-terminus of hnRNP R confirmed depletion of the full-length isoform in motoneurons from *Hnrnpr^tm1a/tm1a^* mice (Figure 4B). Using an antibody against the C-terminus of hnRNP R, we found that the distribution of the hnRNP R-ΔN isoform in knockout motoneurons was similar to the distribution of both isoforms in wildtype motoneurons, with a strong immunosignal in the nucleus and less staining in the cytoplasm (Figure 4B and C). Survival of *Hnrnpr*^*tm1a/tm1a*^ motoneurons cultured for 7 DIV was dependent on the neurotrophic factor BDNF but did not differ from that of wildtype motoneurons (Figure 4D). Quantification of the axon length of motoneurons grown for 7 DIV showed that depletion of hnRNP R-FL had no effect on axon growth (Figure 4E), in contrast to the axon elongation defects observed upon suppression of both hnRNP R isoforms (4) (Supplemental Figure S3A). We also did not observe any changes in growth cone size in motoneurons from *Hnrnpr*^*tm1a/tm1a*^ mice compared to wildtype motoneurons (Figure 4F). Thus, motoneuron development *in vitro* appears unperturbed following loss of hnRNP R-FL.

We previously showed that knockdown of hnRNP R in primary mouse motoneurons reduced the axonal translocation of 7SK, the top RNA interactor of hnRNP R (5). To investigate if depletion of the hnRNP R-FL isoform alone interferes with the axonal localization of 7SK, we performed high-resolution *in situ* hybridization on *Hnrnpr*^*tm1a/tm1a*^ and wildtype motoneurons grown for 6 DIV. Quantification of 7SK punctae revealed that 7SK translocation into axons was not disturbed in motoneurons from *Hnrnpr^tm1a/tm1a^* mice (Figure 4G and H), while it was severely altered when we used lentiviral knockdown of both hnRNP R isoforms (5) (Supplemental Figure S3B and 3C). Taken together, our results suggest that the N-terminal domain of hnRNP R is dispensable for its function in axon elongation and axonal RNA transport in motoneurons.

### hnRNP R binds to chromatin

**Figure 5.**
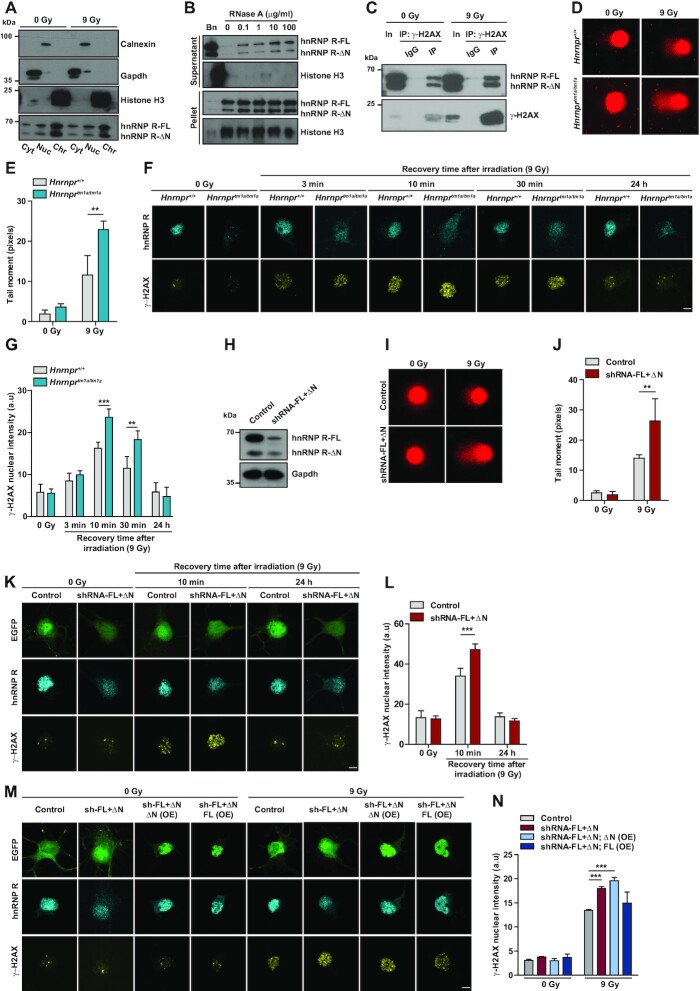
Increased DNA damage and impaired DDR in *Hnrnpr*^*tm1a/tm1a*^ motoneurons. (**A**) Western blot analysis of subcellular fractions from control motoneurons (0 Gy) or motoneurons subjected to γ-irradiation (9 Gy). Motoneurons were fractioned into cytosolic (Cyt), nuclear soluble (Nuc) and chromatin-bound (Chr) fractions. Fractions were probed with the indicated antibodies. (**B**) Western blot analysis of chromatin fractions of NSC-34 cells incubated with Benzonase (Bn) or the indicated amounts of RNase A and separated into supernatant and pellet by centrifugation. (**C**) Immunoprecipitation of γ-H2AX from nuclear fractions of control (0 Gy) or irradiated (9 Gy) NSC-34 cells. Proteins were analyzed by western blot using antibodies against γ-H2AX or hnRNP R. In, input; IP, immunoprecipitation. (**D**) Representative images of alkaline comet assays performed on *Hnrnpr^+^^/+^* and *Hnrnpr^tm1a/tm1a^* motoneurons cultured for 6 DIV under control conditions (0 Gy) and after exposure to γ-irradiation (9 Gy). (**E**) Quantification of comet mean tail moments. Data are mean ± SD (*n* = 3 independent experiments; *N* = 71 nuclei for *Hnrnpr^+^^/+^* (0 Gy), *N* = 55 nuclei for *Hnrnpr^+^^/+^* (9 Gy), *N* = 82 nuclei for *Hnrnpr^tm1a/tm1a^* (0 Gy) and *N* = 76 nuclei for *Hnrnpr^tm1a/tm1a^* (9 Gy)). Statistical analysis was performed using two-way ANOVA followed by Bonferroni post-hoc test; **P ≤ 0.01. (**F**) Representative images of γ-H2AX immunofluorescence staining of *Hnrnpr^+^^/+^* and *Hnrnpr^tm1a/tm1a^* motoneurons under non-irradiated conditions (0 Gy) or after exposure to γ-irradiation (9 Gy) followed by the indicated recovery time. Scale bar: 5 μm. (**G**) Quantification of nuclear γ-H2AX immunostaining shown in (F). Data are mean ± SD (*n* = 4 independent experiments; N = 38 nuclei for *Hnrnpr^+^^/+^* (0 Gy), *N* = 40 nuclei for *Hnrnpr^+^^/+^* (9 Gy, 3 min recovery), *N* = 40 nuclei for *Hnrnpr^+^^/+^* (9 Gy, 10 min recovery), *N* = 41 nuclei for *Hnrnpr^+^^/+^* (9 Gy, 30 min recovery), *N* = 40 nuclei for *Hnrnpr^+^^/+^* (9 Gy, 24 h recovery), *N* = 42 nuclei for *Hnrnpr^tm1a/tm1a^* (0 Gy), *N* = 39 nuclei for *Hnrnpr^tm1a/tm1a^* (9 Gy, 3 min recovery), *N* = 41 nuclei for *Hnrnpr^tm1a/tm1a^* (9 Gy, 10 min recovery), *N* = 40 nuclei for *Hnrnpr^tm1a/tm1a^* (9 Gy, 30 min recovery) and *N* = 39 nuclei for *Hnrnpr^tm1a/tm1a^* (9 Gy, 24 h recovery)). Statistical analysis was performed using two-way ANOVA followed by Bonferroni post-hoc test; **P ≤ 0.01, ***P ≤ 0.001. (**H**) Western blot analysis of hnRNP R levels in cultured motoneurons transduced with control or shRNA against both hnRNP R isoforms (shRNA-FL+ΔN). Gapdh was used as loading control. (**I**) Representative images of alkaline comet assay performed on motoneurons transduced with control and shRNA-FL+ΔN under non-irradiated conditions (0 Gy) or after exposure to γ-irradiation (9 Gy). (**J**) Quantification of comet mean tail moments. Data are mean ± SD (*n* = 3 independent experiments; *N* = 32 nuclei for Control (0 Gy), *N* = 29 nuclei for Control (9 Gy), *N* = 37 nuclei for shRNA-FL+ΔN (0 Gy) and *N* = 30 nuclei for shRNA-FL+ΔN (0 Gy)). Statistical analysis was performed using two-way ANOVA followed by Bonferroni post-hoc test; **P ≤ 0.01. (**K**) Representative images of γ-H2AX immunofluorescence staining of control and shRNA-FL+ΔN-transduced motoneurons under non-irradiated conditions (0 Gy) or after exposure to γ-irradiation (9 Gy) followed by the indicated recovery time. EGFP was used as a marker to identify transduced cells. Scale bar: 5 μm. (**L**) Quantification of nuclear γ-H2AX immunostaining in (K). Data are mean ± SD (*n* = 3 independent experiments; *N* = 34 nuclei for control (0 Gy), *N* = 32 nuclei for control (9 Gy, 10 min recovery), *N* = 22 nuclei for control (9 Gy, 24 h recovery), *N* = 21 nuclei for shRNA-FL+ΔN (0 Gy), *N* = 33 nuclei for shRNA-FL+ΔN (9 Gy, 10 min recovery), *N* = 36 nuclei for shRNA-FL+ΔN (9 Gy, 24 h recovery)). Statistical analysis was performed using two-way ANOVA followed by Bonferroni post-hoc test; ***P ≤ 0.001. (**M**) Representative images of γ-H2AX immunofluorescence staining of motoneurons transduced with control, shRNA-FL+ΔN, shRNA-FL+ΔN; ΔN(OE) or shRNA-FL+ΔN; FL(OE) constructs under non-irradiated conditions (0 Gy) or after exposure to γ-irradiation (9 Gy) followed by 10 min recovery. EGFP was used as a marker to identify cells transduced with the control or shRNA-FL+ΔN construct. The rescue constructs shRNA-FL+ΔN; ΔN(OE) and shRNA-FL+ΔN; FL(OE) expressed EGFP-tagged hnRNP R-ΔN or hnRNP R-FL, respectively, in addition to an shRNA targeting both endogenous hnRNP R isoforms. Scale bar: 5 μm. (**N**) Quantification of nuclear γ-H2AX immunostaining in (M). Data are mean ± SD (*n* = 3 independent experiments; *N* = 25 nuclei for control (0 Gy), *N* = 25 nuclei for control (9 Gy), *N* = 25 nuclei for shRNA-FL+ΔN (0 Gy), *N* = 25 nuclei for shRNA-FL+ΔN (9 Gy), *N* = 25 nuclei for shRNA-FL+ΔN; ΔN(OE) (0 Gy), *N* = 25 nuclei for shRNA-FL+ΔN; ΔN(OE) (9 Gy), *N* = 25 nuclei for shRNA-FL+ΔN; FL(OE) (0 Gy) and *N* = 25 nuclei for shRNA-FL+ΔN; FL(OE) (9 Gy)). Statistical analysis was performed using two-way ANOVA followed by Bonferroni post-hoc test; ***P ≤ 0.001.

After having shown that hnRNP R-ΔN can compensate for loss of hnRNP R-FL with respect to its cytosolic functions, we next focused on its nuclear roles. To do so, we first investigated whether the nuclear pool of hnRNP R is associated with chromatin or with particles that are freely soluble in the nucleosol. For this purpose, we used subcellular fractionation, which allowed us to assess the levels of hnRNP R in the cytosolic fraction (Cyt), the fraction containing organellar and nuclear soluble particles (Nuc), and the chromatin-bound fraction (Chr). The latter was obtained by treating the insoluble nuclear pellet with Benzonase, an endonuclease that degrades both DNA and RNA, to release proteins from chromatin. The effectiveness of the fractionation protocol was confirmed by immunoblot analysis of Gapdh as cytosolic marker for the Cyt fraction, Calnexin as organellar marker for the Nuc fraction, and histone H3 as chromatin marker for the Chr fraction (Figure 5A). Consistent with our immunofluorescence experiments, only a small percentage of hnRNP R was present in the Cyt fraction while most hnRNP R was detectable in the Nuc and Chr fractions. Among these, the majority of hnRNP R was present in the Chr fraction (Figure 5A and Supplemental Figure S4A and S4B).

The enrichment of hnRNP R in the Chr fraction could be the result of its binding to nascent transcripts, which are still tethered to DNA. To address this possibility, we treated the insoluble nuclear pellet with RNase A to release proteins that are bound to chromatin in a RNA-dependent manner (Figure 5B). Following centrifugation, the supernatant contains solubilized proteins, while the pellet contains proteins bound tightly to chromatin. These experiments were performed with the mouse neuronal cell line NSC-34, which has been described previously as having motoneuron-like properties (32). We validated the specificity of the procedure by omission of RNase A, which caused pelleting of hnRNP R and histone H3 into the insoluble fraction, and by treatment with Benzonase, upon which hnRNP R and histone H3 were released into the supernatant. While histone H3 remained in the insoluble fraction with increasing amounts of RNase A, a subset of hnRNP R was released from chromatin into the supernatant. However, even at very high concentration of RNase A (100 μg/ml), a major fraction of hnRNP R remained anchored to chromatin. These results suggest that the association of hnRNP R with chromatin is only partly dependent on RNA and might involve protein contacts.

### Loss of full-length hnRNP R impairs DNA damage repair

Interaction of hnRNP R with chromatin might be mediated through association with histones. In support of this notion, proteomic studies on hepatocellular carcinoma cells have identified hnRNP R as candidate interactor of the histone variant H2AX (33,34). H2AX regulates several processes related to chromatin such as nucleosome formation and chromatin-remodeling, but also plays a major role in DDR following occurrence of double-strand breaks (DSBs) (35). Upon DNA damage, H2AX is rapidly phosphorylated at Ser 139 (γ-H2AX) in the vicinity of DNA breaks, which triggers the recruitment of DNA repair proteins. To investigate whether hnRNP R associates with γ-H2AX, we exposed NSC-34 cells to γ-irradiation (9 Gy) and immunoprecipitated γ-H2AX from nuclear extracts pre-treated with Benzonase. Immunoblot analysis showed that hnRNP R co-precipitated with γ-H2AX after exposure to irradiation (Figure 5C). Our data thus identify γ-H2AX as interaction partner for hnRNP R suggesting that hnRNP R might play a role in DNA repair.

To assess if loss of the hnRNP R-FL isoform impairs DNA damage repair, we exposed *Hnrnpr*^*tm1a/tm1a*^ and *Hnrnpr^+/+^* motoneurons grown for 6 DIV to γ-irradiation (9 Gy) and measured DSBs by comet assay under alkaline conditions. Without irradiation, the mean tail moment did not differ between *Hnrnpr^+/+^*and *Hnrnpr*^*tm1a/tm1a*^ motoneurons and was low due to absence of any overt DNA damage (Figure 5D and E). However, upon exposure to γ-irradiation, nuclei of *Hnrnpr*^*tm1a/tm1a*^ motoneurons showed a significantly increased tail moment compared to nuclei of *Hnrnpr^+/+^*motoneurons (Figure 5D and E), indicating that loss of hnRNP R-FL leads to enhanced DNA damage that cannot be compensated by hnRNP R-ΔN.

To substantiate these findings, we assessed γ-H2AX levels during recovery from γ-irradiation in *Hnrnpr*^*tm1a/tm1a*^ and *Hnrnpr^+/+^* motoneurons. Since quantification of individual γ-H2AX foci was not feasible due to clustering, we measured average fluorescence intensity within cell nuclei. Without irradiation, only very low levels of γ-H2AX were detectable in nuclei of motoneurons of both genotypes (Figure 5F and G). After exposure to 9 Gy of γ-irradiation, the γ-H2AX signal reached a peak at 10 min post-irradiation and then decreased gradually. After 10 and 30 min recovery, we observed an increased γ-H2AX immunosignal in *Hnrnpr*^*tm1a/tm1a*^ compared to *Hnrnpr^+/+^*motoneurons (Figure 5F and G). When we extended the recovery time to 24 h, nuclear γ-H2AX levels returned to their basal state in motoneurons of both genotypes (Figure 5F and G). DSBs and γ-H2AX levels were also increased in *Hnrnpr*^*tm1a/tm1a*^ relative to *Hnrnpr^+/+^*motoneurons 10 min after treatment with 10 μM of the DSB-inducing topoisomerase II inhibitor etoposide for 14 h (Supplemental Figure S4C–F).

Next, we assessed the extent of DNA damage in motoneurons when both hnRNP R isoforms were depleted. For this purpose, we transduced motoneurons with a shRNA targeting both isoforms, leading to a 90% reduction in hnRNP R-FL and 70% reduction in hnRNP R-ΔN isoforms levels (Figure 5H and Supplemental Figure S4G). Following irradiation and recovery for 10 min, motoneurons depleted of both hnRNP R isoforms showed increased comet tail moments (Figure 5I and J) and γ-H2AX levels (Figure 5K and L) compared to control motoneurons. Similarly, motoneurons depleted of both hnRNP R isoforms showed increased γ-H2AX levels 10 min after treatment with 10 μM of etoposide for 14 h (Supplemental Figure S4H and I). Importantly, the extent of DNA damage in hnRNP R knockdown motoneurons relative to controls after 10 min of recovery was similar to the alterations we observed in *Hnrnpr^tm1a/tm1a^* relative to *Hnrnpr^+/+^* motoneurons. This finding suggests that the DNA repair capability of hnRNP R is mostly carried out by the full-length isoform. To further address this possibility, we performed rescue experiments to assess the ability of the hnRNP R-FL or hnRNP R-ΔN isoform to prevent the increase in γ-H2AX levels observed in irradiated motoneurons depleted of both isoforms. We found that while expression of EGFP-tagged hnRNP R-FL resulted in γ-H2AX levels in irradiated hnRNP R-FL+ΔN knockdown motoneurons similar to controls, expression of EGFP-tagged hnRNP R-ΔN did not reduce γ-H2AX levels following depletion of both hnRNP R isoforms (Figure 5M and N). Taken together, these results suggest that loss of hnRNP R-FL delays the repair of DNA breaks, indicating that the N-terminal domain of hnRNP R is required for efficient DDR signaling.

### Yb1 interacts with full-length hnRNP R and regulates DNA repair in motoneurons

**Figure 6.**
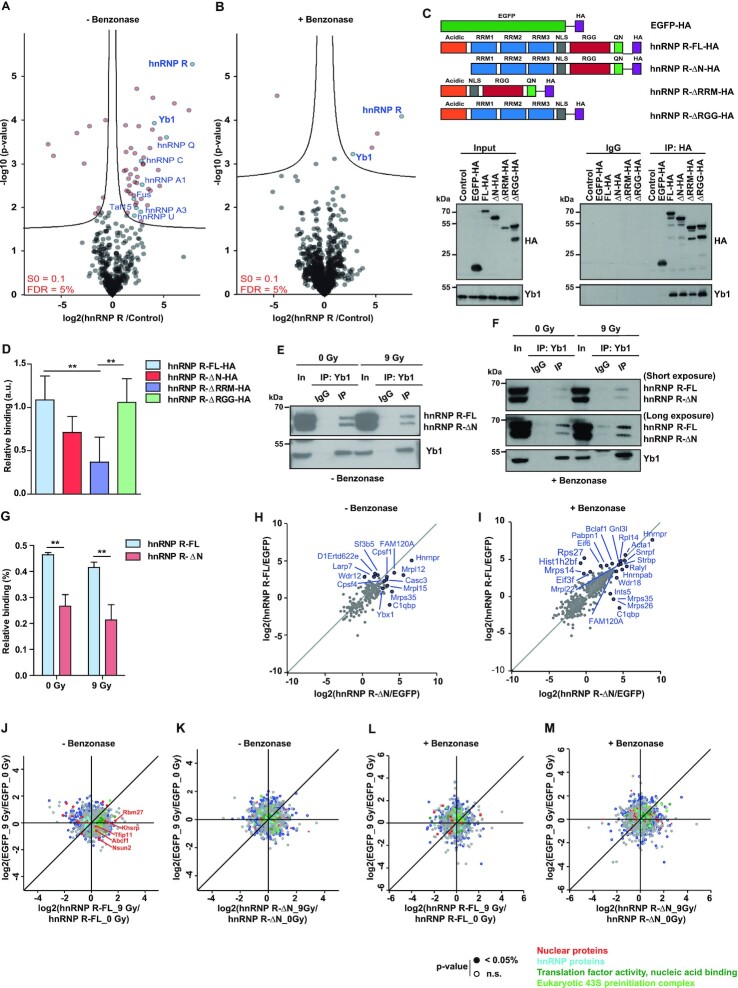
Yb1 shows preferential interaction with the full-length isoform of hnRNP R. (**A, B**) Volcano plots showing proteins identified by mass spectrometry that were significantly enriched after immunoprecipitation with a C-terminal-specific hnRNP R antibody relative to IgG control immunoprecipitations. Experiments were carried out in the absence (A) or presence (B) of Benzonase. Log-transformed p-values (*t* test) associated with individual proteins were plotted against log-transformed fold changes in protein enrichment between hnRNP R and control immunoprecipitations. (**C**) Anti-HA immunoprecipitation from HEK293TN cells transfected with plasmids for expression of HA-tagged hnRNP R isoforms or deletion mutants, or HA-tagged EGFP, or from untransfected cells (control). Proteins were analyzed by western blot with antibodies against Yb1 and HA-tag. In, input; IP, immunoprecipitation. Input represents 5% of the lysate used for immunoprecipitation. (**D**) Quantification of Yb1 co-immunoprecipitation with hnRNP R constructs. Data are mean ± SD (*n* = 5 independent experiments). Statistical analysis was performed using two-way ANOVA followed by Bonferroni post-hoc test; **P ≤ 0.01. (**E, F**) Immunoprecipitation of Yb1 from lysates of non-irradiated (0 Gy) or irradiated (9 Gy) NSC-34 cells in the absence (E) or presence (F) of Benzonase treatment. Proteins were analyzed by Western blot with antibodies against Yb1 and hnRNP R. In, input; IP, immunoprecipitation. Input represents 5% of the lysate used for immunoprecipitation. (**G**) Quantification of hnRNP R isoforms co-immunoprecipitating with Yb1 in the presence of Benzonase (F) normalized to the input. Data are mean ± SD (*n* = 3 independent experiments). Statistical analysis was performed using two-way ANOVA followed by Bonferroni post-hoc test; **P ≤ 0.01. (**H, I**) Scatter plots showing proteins identified by mass spectrometry after immunoprecipitation with anti-HA antibody from HEK293TN transfected with plasmids for expression of hnRNP R-FL-HA, hnRNP R-ΔN-HA or EGFP-HA as control. Data are shown as log-transformed fold changes in protein enrichment between hnRNP R-FL-HA or hnRNP R-ΔN-HA and EGFP-HA immunoprecipitations, carried out in the absence (H) or presence (I) of Benzonase. (**J, K**) Scatter plots showing proteins identified by mass spectrometry after immunoprecipitation with anti-HA antibody from HEK293TN expressing hnRNP R-FL-HA (J), hnRNP R-ΔN-HA (K) or EGFP-HA under non-irradiated (0 Gy) or after exposure to irradiation (9 Gy). Experiments were carried out in the absence of Benzonase. Log-transformed fold changes in protein enrichment in EGFP-HA immunoprecipitations under non-irradiated (0 Gy) and irradiated (9 Gy) conditions were plotted against log-transformed fold changes in protein enrichment in hnRNP R-FL-HA (J) or hnRNP R-ΔN-HA (K) immunoprecipitations under non-irradiated (0 Gy) and irradiated (9 Gy) conditions. Functional annotations of proteins are colour-coded. (**L, M**) Same as (J, K) but in the presence of Benzonase.

To obtain mechanistic insights into the role of hnRNP R in DNA damage repair, we investigated the protein interactome of hnRNP R in motoneurons. We immunopurified hnRNP R complexes from cultured embryonic motoneurons using an antibody against the C-terminus of hnRNP R and identified proteins by mass spectrometry. We detected 77 proteins significantly enriched in the hnRNP R immunoprecipitate relative to IgG control (Figure 6A and Supplemental Table S1). Among these, hnRNP R was the top hit indicating the specificity of the procedure. Analysis of hnRNP R interactors revealed several RBPs in agreement with its role as mRNP component. Among these, we detected hnRNP U, Taf15, hnRNP A1, hnRNP A3, hnRNP Q, hnRNP C and Fus, which have previously been found to associate with hnRNP R (10,36,37).

To identify protein interactions that are not mediated via nucleic acids, we additionally performed the immunoprecipitation in the presence of Benzonase and analysed the interactors by mass spectrometry. The number of proteins associated with hnRNP R after Benzonase treatment was much lower compared to when nucleic acids were left intact (Figure 6B). Nevertheless, under both conditions we found Msy1, also known as Y-box binding protein 1 (Yb1), as major interactor of hnRNP R (Figure 6A and B). Yb1 is a RNA/DNA-binding protein involved in a wide variety of cellular functions in both the nucleus and the cytoplasm, including DNA repair (38). In order to investigate which region of hnRNP R is involved in binding to Yb1, we expressed HA-tagged hnRNP R-FL, hnRNP R-ΔN, and mutants with deletion of the RRM (hnRNP R-ΔRRM) or RGG domain (hnRNP R-ΔRGG) in HEK293TN cells. The Nuclear Localization Signal (NLS) was preserved in all constructs. As control, we used untransfected cells and cells transfected with HA-tagged EGFP. Following pulldown with anti-HA beads, Yb1 association with hnRNP R was reduced slightly in the absence of the N-terminus, but more prominently in the absence of the RRM domain. Thus, its N-terminal acidic domain and its RNA-binding capability contribute to the association of hnRNP R with Yb1 (Figure 6C and D).

To investigate whether the interaction between hnRNP R and Yb1 is regulated by irradiation, we immunoprecipitated Yb1 in the presence or absence of Benzonase from irradiated NSC-34 cells. In agreement with our proteomics results, both hnRNP R isoforms co-precipitated with Yb1 in the presence as well as in the absence of Benzonase treatment (Figure 6E and F). Additionally, while both hnRNP R isoforms co-precipitated with Yb1 independent of irradiation exposure (Figure 6E and F), we observed that Yb1 displayed a 1.7-fold preference for the interaction with hnRNP R-FL, compared to hnRNP R-ΔN (Figure 6G).

We next sought to investigate the specific protein interactome of each hnRNP R isoform. For this purpose, we expressed HA-tagged hnRNP R-FL, hnRNP R-ΔN and EGFP as control in HEK293TN cells, followed by purification with anti-HA beads and analysis of interacting proteins by mass spectrometry. We first determined the interactomes in the absence or presence of Benzonase. As expected, hnRNP R was the most abundant protein in the hnRNP R-FL and hnRNP R-ΔN immunoprecipitates (Figure 6H and I and Supplemental Table S2). We observed that while both isoforms share some common interactors, each of the isoforms also possesses its own repertoire of binding partners (Supplemental Figure S7). For example, we found that LARP7, a core interactor of 7SK, was more strongly enriched for hnRNP R-FL. Its interaction with hnRNP R-FL was RNA-dependent in agreement with a previous study (39). Notably, while Yb1 was detectable among co-immunoprecipitated proteins, we did not observe an enrichment in the hnRNP R-FL immunoprecipitates relative to hnRNP R-ΔN. This might be due to differences in purification efficiency between hnRNP R-FL and hnRNP R-ΔN or the use HEK293TN cells. To gain further insights into the involvement of each of hnRNP R isoform in DDR, we performed mass spectrometry analysis following pulldown of hnRNP R isoforms from irradiated cells. Intriguingly, we observed that most of the interactome changes upon irradiation occurred with the hnRNP R-FL isoform, while hnRNP R-ΔN seems to be insensitive to irradiation (Figure 6J–M). Specifically, we observed less interaction of hnRNP R-FL with hnRNP proteins and additional associations with nuclear proteins upon irradiation (Figure 6J). Among these was Khsrp, a protein thought to be involved in DDR (40).

**Figure 7.**
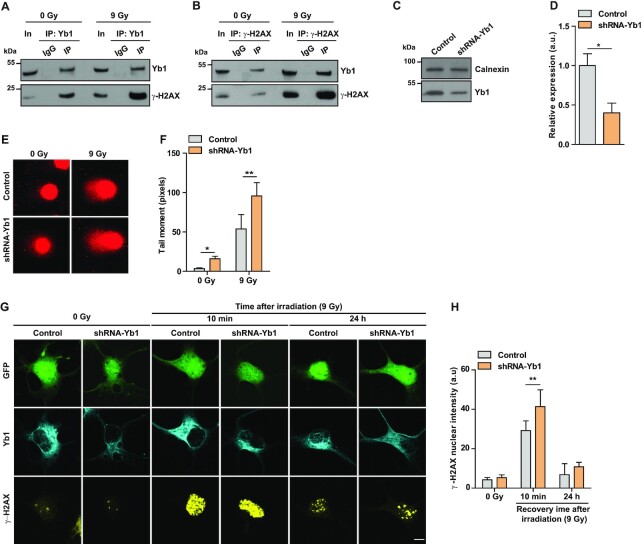
Yb1 knockdown results in DNA damage defects in motoneurons. (**A, B**) Immunoprecipitation of Yb1 (A) or γ-H2AX (B) performed on whole lysates of non-irradiated (0 Gy) or irradiated (9 Gy) NSC-34 cells. Proteins were analyzed by western blot with antibodies against γ-H2AX and Yb1. In, input; IP, immunoprecipitation. (**C**) Western blot analysis of Yb1 levels in cultured motoneurons transduced with control or shRNA-Yb1. Calnexin was used as loading control. (**D**) Quantification of western blot data shown in (C). Data are mean ± SD (*n* = 3 independent experiments). Statistical analysis was performed using unpaired *t*-test; *P ≤ 0.05. (**E**) Representative images of alkaline comet assay performed on control and shRNA-Yb1-transduced motoneurons under non-irradiated conditions (0 Gy) or after exposure to γ-irradiation (9 Gy). (**F**) Quantification of comet mean tail moments. Data are mean ± SD (*n* = 3 independent experiments; *N* = 31 nuclei for control (0 Gy), *N* = 34 nuclei for control (9 Gy), *N* = 33 nuclei for shRNA-Yb1 (0 Gy) and *N* = 33 nuclei for shRNA-Yb1 (9 Gy)). Statistical analysis was performed using two-way ANOVA followed by Bonferroni post-hoc test; *P ≤ 0.05, **P ≤ 0.01. (**G**) Representative images of γ-H2AX immunofluorescence staining of control and shRNA-Yb1-transduced motoneurons under non-irradiated conditions (0 Gy) or after exposure to γ-irradiation (9 Gy) followed by the indicated recovery times. EGFP was used as a marker to identify transduced cells. Scale bar: 5 μm. (**H**) Quantification of nuclear γ-H2AX immunostaining in (G). Data are mean ± SD (*n* = 4 independent experiments; *N* = 42 nuclei for control (0 Gy), *N* = 40 nuclei for control (9 Gy,10 min recovery), *N* = 18 nuclei for control (9 Gy, 24 h recovery), *N* = 34 nuclei for shRNA-Yb1 (0 Gy), *N* = 43 nuclei for shRNA-Yb1 (9 Gy, 10 min recovery) and *N* = 31 nuclei for shRNA-Yb1 (9 Gy, 24 h recovery)). Statistical analysis was performed using two-way ANOVA followed by Bonferroni post-hoc test; **P ≤ 0.01.

Our data show that loss of hnRNP R-FL disrupts DNA damage repair in motoneurons (Figure 5D–G). Given that hnRNP R associates with γ-H2AX upon DNA damage (Figure 5C), we next asked whether Yb1 interacts with γ-H2AX upon irradiation. We found that immunoprecipitation of Yb1 from irradiated NSC-34 cells co-precipitated higher amounts of γ-H2AX compared to non-irradiated cells (Figure 7A). Immunoprecipitation with an antibody against γ-H2AX confirmed its interaction with Yb1 (Figure 7B). Taken together, our data indicate that DNA damage induces recruitment of hnRNP R/Yb1 complexes to sites of DNA damage through interaction with γ-H2AX.

To investigate whether Yb1 has DNA damage-associated functions in motoneurons, we knocked down Yb1 by lentiviral transduction with a shRNA (Figure 7C and D) and used the comet assay to assess DNA breaks in non-irradiated and irradiated control and Yb1 knockdown motoneurons. Without irradiation, nuclei of control motoneurons showed only very few comet tails indicative of low levels of DNA breaks (Figure 7E and F). Compared to controls, nuclei of non-irradiated Yb1 knockdown motoneurons contained higher levels of DNA breaks indicating that depletion of Yb1 affects DNA repair mechanisms already at basal levels. The increased level of DNA damage upon Yb1 knockdown was also detectable after irradiation, when nuclei of Yb1 knockdown motoneurons showed significantly increased tail moments compared to controls (Figure 7E and F). To corroborate these findings, we assessed γ-H2AX levels in Yb1 knockdown and control motoneurons by immunostaining. In control motoneurons, endogenous Yb1 was detectable throughout the cytoplasm and axons of motoneurons (Figure 7G). In Yb1 shRNA-transduced motoneurons, the Yb1 immunosignal was reduced consistent with the protein knockdown detected by immunoblot analysis (Figure 7G). In the absence of irradiation, γ-H2AX immunoreactivity was low in both control and Yb1 shRNA-treated cells (Figure 7G and H). Following exposure to γ-irradiation and recovery for 10 min, the γ-H2AX immunoreactivity was increased in both control and Yb1 knockdown motoneurons. Compared to controls, the increase in γ-H2AX immunoreactivity was higher in Yb1-depleted motoneurons (Figure 7G and H). After 24 h of recovery, γ-H2AX levels returned to near-basal levels in both control and Yb1 knockdown motoneurons (Figure 7G and H). Taken together, these data indicate that, similar to loss of hnRNP R-FL, Yb1 depletion affects DDR signaling in motoneurons.

### DNA damage enhances chromatin binding of Yb1 in an hnRNP R-dependent manner

**Figure 8.**
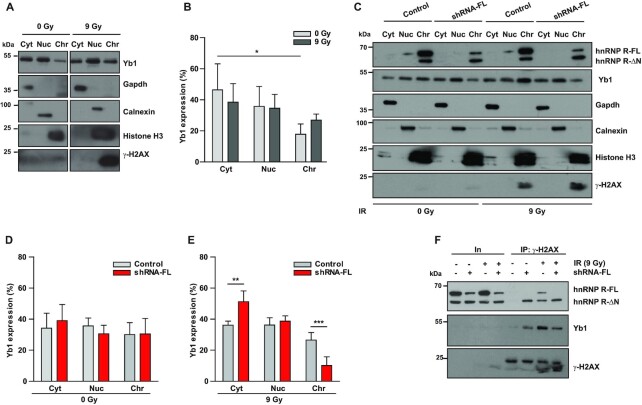
Recruitment of Yb1 to chromatin in response to DNA damage is impaired in the absence of the full-length hnRNP R isoform. (**A**) Non-irradiated NSC-34 cells (0 Gy) or NSC-34 cells subjected to γ-irradiation (9 Gy) were separated into cytosolic (Cyt), nuclear soluble (Nuc) and chromatin-bound (Chr) fractions. Fractions were analyzed by western blot with the indicated antibodies. (**B**) Quantification of western blot data shown in (A). The expression of Yb1 in each fraction is presented as the percentage of total. Data are mean ± SD (*n* = 3 independent experiments). Statistical analysis was performed using two-way ANOVA followed by Bonferroni post-hoc test; *P ≤ 0.05. (**C**) Non-irradiated (0 Gy) or irradiated (9 Gy) NSC-34 cells transduced with control lentivirus or lentivirus expressing shRNA-FL for knockdown of full-length hnRNP R were fractionated and analyzed by western blot with the indicated antibodies. (**D, E**) Quantification of the Yb1 Western blot data shown in (C) for control conditions (0 Gy) (D) and after exposure to γ-irradiation (9 Gy) (E). The expression of Yb1 in each fraction is presented as the percentage of total. Data are mean ± SD (*n* = 3 independent experiments). Statistical analysis was performed using two-way ANOVA followed by Bonferroni post-hoc test; **P ≤ 0.01, ***P ≤ 0.001. (**F**) Immunoprecipitation of γ-H2AX from lysates of non-irradiated (0 Gy) or irradiated (9 Gy) NSC-34 cells transduced with control lentivirus or lentivirus expressing shRNA-FL for knockdown of full-length hnRNP R. Proteins were analyzed by Western blot with the indicated antibodies. In, input; IP, immunoprecipitation.

Next, we investigated whether hnRNP R regulates the chromatin association of Yb1 in response to irradiation by performing subcellular fractionation of irradiated NSC-34 cells. We found that chromatin binding of Yb1 was enhanced upon exposure to γ-irradiation, coinciding with the increased levels of γ-H2AX (Figure 8A and B). Given that Yb1 interacts with hnRNP R, and that DNA repair is impaired in *Hnrnpr*^*tm1a/tm1a*^ motoneurons, we next asked whether hnRNP R-FL regulates Yb1 recruitment to chromatin. Since we could not perform subcellular fractionation on motoneurons derived from single embryos of *Hnrnpr*^*tm1a/tm1a*^ mice due to limited material, we used NSC-34 cells transduced with a shRNA targeting hnRNP R-FL (shRNA-FL-C, Figure 3C) and performed subcellular fractionation with and without exposure to irradiation. Under non-irradiated conditions, the distribution of Yb1 was similar between control and hnRNP R-FL knockdown motoneurons (Figure 8C and D). However, upon exposure to irradiation we observed that Yb1 levels were increased in the cytosol of hnRNP R-FL knockdown motoneurons compared to control motoneurons (Figure 8C and E). At the same time, association of Yb1 with chromatin upon exposure to irradiation was impaired by knockdown of hnRNP R-FL (Figure 8C and E). Since Yb1 is a RNA-binding protein, we tested the possibility that an excess of *Hnrnpr* mRNA lacking exon 2, as occurs following knockdown of hnRNP R-FL, prevents Yb1 recruitment into the nucleus following irradiation. We performed Yb1 RNA immunoprecipitation (RIP) from NSC-34 cells under control conditions and after exposure to irradiation (Supplemental Figure S5). As positive control, we observed an enrichment of *Eef2* mRNA in the Yb1 immunoprecipitate, which is a transcript known to bind Yb1 (41). For *Hnrnpr* mRNA, we did not observe any difference in the binding capacity between the *Hnrnpr-FL* and *Hnrnpr-ΔN* transcript to Yb1. Additionally, after irradiation we did not detect an increased binding of Yb1 to the *Hnrnpr-ΔN* transcript, which argues against a potential role for this transcript in preventing Yb1 recruitment to chromatin upon DNA damage exposure. To further address how the interaction between hnRNP R and Yb1 regulates DNA repair, we selectively knocked down the full-length isoform of hnRNP R and investigated the association of Yb1 with γ-H2AX. We found that while Yb1 association with γ-H2AX was strongly enhanced upon irradiation in control cells, depletion of hnRNP R-FL prevented irradiation-induced Yb1 binding to γ-H2AX (Figure 8F). Taken together, these results suggest that the hnRNP R-FL is required for chromatin binding and anchoring of Yb1 and its binding to γ-H2AX after exposure to irradiation.

## DISCUSSION

Long-lived postmitotic neurons are particularly vulnerable to various forms of DNA damage and depend on highly efficient DNA repair mechanisms to maintain their genomic integrity. There is increasing evidence that failure to repair defects and the resulting accumulation of unrepaired DNA lesions contribute to neurodegenerative disorders such as SMA and ALS (42–44). However, the link between DNA repair deficiency and degenerative diseases is still limited, raising the need to identify the components involved in this process. Several studies have implicated RBPs in DDR signaling. The RBP hnRNP R is enriched in the nervous system and has a diverse range of functions in regulating gene expression (45–48). Here, we show that exon 2 of the *Hnrnpr* transcript undergoes alternative splicing, producing two protein isoforms that differ at their N-terminus. We used a mouse model with selective depletion of the hnRNP R-FL isoform to show that it has a specific role in DNA damage repair. We found that the function of hnRNP R in DNA repair involves Yb1, whose association with chromatin and γ-H2AX upon DNA damage was dependent on the full-length isoform of hnRNP R. Thus, our data reveal a specific function of a RBP splice isoform in genome maintenance and repair.

Our study shows that depletion of the full-length isoform of hnRNP R *in vivo* as well as *in vitro* results in a compensatory upregulation of the N-terminally truncated hnRNP R-ΔN isoform in neuronal tissues (brain and spinal cord). This increase is likely due to regulatory mechanisms at the post-transcriptional level, since we did not observe any difference at the pre-mRNA level in *Hnrnpr^tm1a/tm1a^* relative to wildtype mice. Such mechanisms might involve autoregulation by binding of hnRNP R to its own pre-mRNA as has been shown for other RBPs (15,49). Alternatively, regulation of other splice factors by hnRNP R might affect *Hnrnpr* exon 2 splicing. For example, it has been shown that knockdown of ADAR, an adenosine deaminase that mediates A-to-I editing, results in the upregulation of *HNRNPR* exon 2 inclusion (50). Importantly, we found that the inclusion of exon 2 is particularly high in the nervous system suggesting that neuron-specific mechanisms might contribute to *Hnrnpr* alternative splicing.

Our RNA and protein analysis showed that the full-length protein is the predominant isoform of hnRNP R in the nervous system suggesting that it has important functions in neurons. Knockdown of both hnRNP R isoforms has previously been shown to affect axon growth and axonal RNA transport (4,6). However, selective depletion of hnRNP R-FL in *Hnrnpr^tm1a/tm1a^* mice did not affect axon growth or axonal RNA transport indicating that the N-terminally truncated isoform can compensate for absence of the full-length protein in these processes. While motoneuron development appears to depend on total levels of hnRNP R, we uncovered a new role of hnRNP R in DDR that is specifically associated with the hnRNP R-FL isoform. We show that motoneurons of mice deficient for hnRNP R-FL exhibit delayed DNA damage repair, as seen by increased γ-H2AX immunoreactivity and increased tail moments in the comet assay. Conspicuously, we detected high levels of the hnRNP R-FL isoform in the nervous system while it was present at much lower levels in other tissues such as liver. This suggests that neurons in particular require hnRNP R-dependent DNA damage repair mechanisms. One possible reason for this could be that neurons are postmitotic and thus particularly depend on efficient DNA repair mechanisms compared to fast regenerating tissues. In support of this notion, DNA damage repair seems to occur less efficiently in liver cells compared to motoneurons (Supplemental Figure S6). Thus, our finding that the hnRNP R-FL isoform has a function in DDR could potentially explain its abundant expression in the developing nervous system.

Proteomic analysis of the hnRNP R interactome revealed the RNA/DNA-binding protein Yb1 as a major interactor. Previous studies found that Yb1 plays a major role in different types of DNA damage repair (51–55), interacting with proteins involved in DSB repair like WRN (56) and Ku80 (54). We now provide evidence that the function of hnRNP R-FL in DNA damage repair involves Yb1. We show that Yb1 knockdown in motoneurons phenocopies the impaired DDR we observed for loss of hnRNP R-FL. Furthermore, we found that Yb1 preferentially binds to hnRNP R-FL compared to hnRNP R-ΔN in NSC-34 cells. Additionally, we demonstrate that DNA damage enhances Yb1 binding to chromatin, and that this translocation is abolished when hnRNP R-FL is depleted. We found that even though the interaction between hnRNP R and Yb1 is not enhanced after exposure to γ-irradiation, their association with γ-H2AX is increased upon DNA damage. This finding points towards the possibility that chromatin-associated Yb1/hnRNP R complexes relocate to γ-H2AX foci at DNA damage sites in agreement with the notion that H2AX phosphorylation serves as an epigenetic signal to orchestrate the ordered recruitment of downstream DDR proteins (35). This notion is supported by our observation that the interaction of Yb1 with γ-H2AX was diminished upon knockdown of hnRNP R-FL. It is possible that the N-terminal acidic domain of hnRNP R enhances the affinity of Yb1 for chromatin by stabilizing complexes between Yb1 and other proteins at DNA damage sites. Alternatively, the N-terminus of hnRNP R might be required to liberate Yb1 from complexes in the cytoplasm thus allowing it to bind to chromatin directly or indirectly. Intriguingly, interactome analysis shows that only the full-length isoform loses interaction with hnRNP proteins upon irradiation, which could reflect a shift in its function as a mRNP component towards increased interaction with nuclear proteins.

In conclusion, our study suggests a novel role for hnRNP R in the DDR in post-mitotic motoneurons. Research over the past years has identified several RBPs, many of them involved in motoneuron diseases, that function in DNA damage repair (57). Loss of nuclear Fus results in DNA nick ligation defects in motoneurons due to reduced recruitment of XRCC1/LigIII to DNA strand breaks (58). TDP-43 interacts with key proteins involved in DNA damage repair including pATM, and p53BP1 (59). Chronic low levels of SMN results in increased RNA–DNA hybrids (R-loops) and DSBs (60,61). Given that hnRNP R interacts with several proteins involved in motoneuron diseases, and that aggregates of hnRNP R have been detected in FTLD (13), it thus remains possible that impaired DNA damage repair through mechanisms that involve dysfunction of hnRNP R might contribute to the pathology underlying certain forms of neurodegenerative disorders.

## DATA AVAILABILITY

Proteomic datasets including annotated MS/MS datasets were uploaded to the ProteomeXchange Consortium via the PRIDE database, and then assigned the dataset identifier PXD022467 and PXD026407.

## Supplementary Material

gkab1120_Supplemental_Files

## Notes

Present address: Hanaa Ghanawi, Department of Pharmacy, Center for Drug Research, Ludwig-Maximilians-Universität München, Munich 81377, Germany.

Present address: Daniel Hornburg, Seer, Inc., Redwood City, CA 94065, USA.

Present address: Rajeeve Sivadasan, Department of Neurobiology, Rajiv Gandhi Centre for Biotechnology (RGCB), Thycaud Post, Poojappura, Thiruvananthapuram 695 014, Kerala, India.

Present address: Carsten Drepper, Department of Child and Adolescent Psychiatry, Psychosomatics and Psychotherapy, University of Wuerzburg, Wuerzburg 97080, Germany.

Present address: Felix Meissner, Department for Systems Immunology & Proteomics, Institute of Innate Immunity, University Hospitals, University of Bonn, Bonn 53127, Germany.

## References

[1] Geuens T. , Bouhy D., Timmerman V. The hnRNP family: insights into their role in health and disease. Hum. Genet. 2016; 135:851–867. 27215579 10.1007/s00439-016-1683-5 PMC4947485

[2] Han S.P. , Tang Y.H., Smith R. Functional diversity of the hnRNPs: past, present and perspectives. Biochem. J. 2010; 430:379–392. 20795951 10.1042/BJ20100396

[3] Rossoll W. , Kroning A.K., Ohndorf U.M., Steegborn C., Jablonka S., Sendtner M. Specific interaction of Smn, the spinal muscular atrophy determining gene product, with hnRNP-R and gry-rbp/hnRNP-Q: a role for Smn in RNA processing in motor axons?. Hum. Mol. Genet. 2002; 11:93–105. 11773003 10.1093/hmg/11.1.93

[4] Glinka M. , Herrmann T., Funk N., Havlicek S., Rossoll W., Winkler C., Sendtner M. The heterogeneous nuclear ribonucleoprotein-R is necessary for axonal β-actin mRNA translocation in spinal motor neurons. Hum. Mol. Genet. 2010; 19:1951–1966. 20167579 10.1093/hmg/ddq073

[5] Briese M. , Saal-Bauernschubert L., Ji C., Moradi M., Ghanawi H., Uhl M., Appenzeller S., Backofen R., Sendtner M. hnRNP R and its main interactor, the noncoding RNA 7SK, coregulate the axonal transcriptome of motoneurons. Proc. Natl. Acad. Sci. U.S.A. 2018; 115:E2859–E2868. 29507242 10.1073/pnas.1721670115 PMC5866599

[6] Rossoll W. , Jablonka S., Andreassi C., Kröning A.-K., Karle K., Monani U.R., Sendtner M. Smn, the spinal muscular atrophy-determining gene product, modulates axon growth and localization of beta-actin mRNA in growth cones of motoneurons. J. Cell Biol. 2003; 163:801–812. 14623865 10.1083/jcb.200304128 PMC2173668

[7] Dombert B. , Sivadasan R., Simon C.M., Jablonka S., Sendtner M. Presynaptic localization of Smn and hnRNP R in axon terminals of embryonic and postnatal mouse motoneurons. PLoS One. 2014; 9:e110846. 25338097 10.1371/journal.pone.0110846 PMC4206449

[8] Mourelatos Z. , Abel L., Yong J., Kataoka N., Dreyfuss G. SMN interacts with a novel family of hnRNP and spliceosomal proteins. EMBO J. 2001; 20:5443–5452. 11574476 10.1093/emboj/20.19.5443 PMC125643

[9] Rossoll W. , Kröning A.-K., Ohndorf U.-M., Steegborn C., Jablonka S., Sendtner M. Specific interaction of Smn, the spinal muscular atrophy determining gene product, with hnRNP-R and gry-rbp/hnRNP-Q: a role for Smn in RNA processing in motor axons?. Hum. Mol. Genet. 2002; 11:93–105. 11773003 10.1093/hmg/11.1.93

[10] Chi B. , O’Connell J.D., Yamazaki T., Gangopadhyay J., Gygi S.P., Reed R. Interactome analyses revealed that the U1 snRNP machinery overlaps extensively with the RNAP II machinery and contains multiple ALS/SMA-causative proteins. Sci. Rep. 2018; 8:8755. 29884807 10.1038/s41598-018-27136-3 PMC5993797

[11] Kamelgarn M. , Chen J., Kuang L., Arenas A., Zhai J., Zhu H., Gal J. Proteomic analysis of FUS interacting proteins provides insights into FUS function and its role in ALS. Biochim. Biophys. Acta. 2016; 1862:2004–2014. 27460707 10.1016/j.bbadis.2016.07.015 PMC5055831

[12] Freibaum B.D. , Chitta R.K., High A.A., Taylor J.P. Global analysis of TDP-43 interacting proteins reveals strong association with RNA splicing and translation machinery. J. Proteome Res. 2010; 9:1104–1120. 20020773 10.1021/pr901076y PMC2897173

[13] Gittings L.M. , Foti S.C., Benson B.C., Gami-Patel P., Isaacs A.M., Lashley T. Heterogeneous nuclear ribonucleoproteins R and Q accumulate in pathological inclusions in FTLD-FUS. Acta Neuropathol. Commun. 2019; 7:18. 30755280 10.1186/s40478-019-0673-y PMC6371513

[14] Duijkers F.A. , McDonald A., Janssens G.E., Lezzerini M., Jongejan A., van Koningsbruggen S., Leeuwenburgh-Pronk W.G., Wlodarski M.W., Moutton S., Tran-Mau-Them F. et al. HNRNPR variants that impair homeobox gene expression drive developmental disorders in humans. Am. J. Hum. Genet. 2019; 104:1040–1059. 31079900 10.1016/j.ajhg.2019.03.024 PMC6556882

[15] Huelga Stephanie C. , Vu Anthony Q., Arnold Justin D., Liang Tiffany Y., Liu Patrick P., Yan Bernice Y., Donohue John P., Shiue L., Hoon S., Brenner S. et al. Integrative genome-wide analysis reveals cooperative regulation of alternative splicing by hnRNP proteins. Cell Rep. 2012; 1:167–178. 22574288 10.1016/j.celrep.2012.02.001 PMC3345519

[16] Skarnes W.C. , Rosen B., West A.P., Koutsourakis M., Bushell W., Iyer V., Mujica A.O., Thomas M., Harrow J., Cox T. et al. A conditional knockout resource for the genome-wide study of mouse gene function. Nature. 2011; 474:337–342. 21677750 10.1038/nature10163 PMC3572410

[17] Wiese S. , Herrmann T., Drepper C., Jablonka S., Funk N., Klausmeyer A., Rogers M.L., Rush R., Sendtner M. Isolation and enrichment of embryonic mouse motoneurons from the lumbar spinal cord of individual mouse embryos. Nat. Protoc. 2010; 5:31–38. 20057379 10.1038/nprot.2009.193

[18] Untergasser A. , Cutcutache I., Koressaar T., Ye J., Faircloth B.C., Remm M., Rozen S.G. Primer3–new capabilities and interfaces. Nucleic Acids Res. 2012; 40:e115. 22730293 10.1093/nar/gks596 PMC3424584

[19] Schindelin J. , Arganda-Carreras I., Frise E., Kaynig V., Longair M., Pietzsch T., Preibisch S., Rueden C., Saalfeld S., Schmid B. et al. Fiji: an open-source platform for biological-image analysis. Nat. Methods. 2012; 9:676–682. 22743772 10.1038/nmeth.2019 PMC3855844

[20] Rehberg M. , Lepier A., Solchenberger B., Osten P., Blum R. A new non-disruptive strategy to target calcium indicator dyes to the endoplasmic reticulum. Cell Calcium. 2008; 44:386–399. 19230142 10.1016/j.ceca.2008.02.002

[21] Holden P. , Horton W.A. Crude subcellular fractionation of cultured mammalian cell lines. BMC Research Notes. 2009; 2:243. 20003239 10.1186/1756-0500-2-243 PMC2802353

[22] Yang L. , Gal J., Chen J., Zhu H. Self-assembled FUS binds active chromatin and regulates gene transcription. Proc. Natl. Acad. Sci. U.S.A. 2014; 111:17809–17814. 25453086 10.1073/pnas.1414004111 PMC4273402

[23] Olive P.L. , Banáth J.P. The comet assay: a method to measure DNA damage in individual cells. Nat. Protoc. 2006; 1:23–29. 17406208 10.1038/nprot.2006.5

[24] Gyori B.M. , Venkatachalam G., Thiagarajan P.S., Hsu D., Clement M.-V. OpenComet: An automated tool for comet assay image analysis. Redox. Biol. 2014; 2:457–465. 24624335 10.1016/j.redox.2013.12.020 PMC3949099

[25] Rappsilber J. , Mann M., Ishihama Y. Protocol for micro-purification, enrichment, pre-fractionation and storage of peptides for proteomics using StageTips. Nat. Protoc. 2007; 2:1896–1906. 17703201 10.1038/nprot.2007.261

[26] Cox J. , Mann M. MaxQuant enables high peptide identification rates, individualized p.p.b.-range mass accuracies and proteome-wide protein quantification. Nat. Biotechnol. 2008; 26:1367–1372. 19029910 10.1038/nbt.1511

[27] Cox J. , Mann M. Quantitative, high-resolution proteomics for data-driven systems biology. Annu. Rev. Biochem. 2011; 80:273–299. 21548781 10.1146/annurev-biochem-061308-093216

[28] Hornburg D. , Drepper C., Butter F., Meissner F., Sendtner M., Mann M. Deep proteomic evaluation of primary and cell line motoneuron disease models delineates major differences in neuronal characteristics. Mol. Cell. Proteomics. 2014; 13:3410–3420. 25193168 10.1074/mcp.M113.037291 PMC4256493

[29] Cox J. , Hein M.Y., Luber C.A., Paron I., Nagaraj N., Mann M. Accurate proteome-wide label-free quantification by delayed normalization and maximal peptide ratio extraction, termed MaxLFQ. Mol. Cell. Proteomics. 2014; 13:2513–2526. 24942700 10.1074/mcp.M113.031591 PMC4159666

[30] Tusher V.G. , Tibshirani R., Chu G. Significance analysis of microarrays applied to the ionizing radiation response. Proc. Natl. Acad. Sci. U.S.A. 2001; 98:5116–5121. 11309499 10.1073/pnas.091062498 PMC33173

[31] Hassfeld W. , Chan E.K., Mathison D.A., Portman D., Dreyfuss G., Steiner G., Tan E.M. Molecular definition of heterogeneous nuclear ribonucleoprotein R (hnRNP R) using autoimmune antibody: immunological relationship with hnRNP P. Nucleic Acids Res. 1998; 26:439–445. 9421497 10.1093/nar/26.2.439 PMC147279

[32] Cashman N.R. , Durham H.D., Blusztajn J.K., Oda K., Tabira T., Shaw I.T., Dahrouge S., Antel J.P. Neuroblastoma× spinal cord (NSC) hybrid cell lines resemble developing motor neurons. Dev. Dyn. 1992; 194:209–221. 1467557 10.1002/aja.1001940306

[33] Du Y.C. , Gu S., Zhou J., Wang T., Cai H., Macinnes M.A., Bradbury E.M., Chen X. The dynamic alterations of H2AX complex during DNA repair detected by a proteomic approach reveal the critical roles of Ca(2+)/calmodulin in the ionizing radiation-induced cell cycle arrest. Mol. Cell. Proteomics. 2006; 5:1033–1044. 16522924 10.1074/mcp.M500327-MCP200

[34] Yang X. , Zou P., Yao J., Yun D., Bao H., Du R., Long J., Chen X. Proteomic dissection of cell type-specific H2AX-interacting protein complex associated with hepatocellular carcinoma. J. Proteome Res. 2010; 9:1402–1415. 20000738 10.1021/pr900932y PMC3670604

[35] Mah L.J. , El-Osta A., Karagiannis T.C. γH2AX: a sensitive molecular marker of DNA damage and repair. Leukemia. 2010; 24:679–686. 20130602 10.1038/leu.2010.6

[36] Hein Marco Y. , Hubner Nina C., Poser I., Cox J., Nagaraj N., Toyoda Y., Gak Igor A., Weisswange I., Mansfeld J., Buchholz F. et al. A human interactome in three quantitative dimensions organized by stoichiometries and abundances. Cell. 2015; 163:712–723. 26496610 10.1016/j.cell.2015.09.053

[37] Stark C. , Breitkreutz B.J., Reguly T., Boucher L., Breitkreutz A., Tyers M. BioGRID: a general repository for interaction datasets. Nucleic Acids Res. 2006; 34:D535–D539. 16381927 10.1093/nar/gkj109 PMC1347471

[38] Lyabin D.N. , Eliseeva I.A., Ovchinnikov L.P. YB-1 protein: functions and regulation. WIREs RNA. 2014; 5:95–110. 24217978 10.1002/wrna.1200

[39] Ji C. , Bader J., Ramanathan P., Hennlein L., Meissner F., Jablonka S., Mann M., Fischer U., Sendtner M., Briese M. Interaction of 7SK with the Smn complex modulates snRNP production. Nat. Commun. 2021; 12:1278. 33627647 10.1038/s41467-021-21529-1 PMC7904863

[40] Briata P. , Bordo D., Puppo M., Gorlero F., Rossi M., Perrone-Bizzozero N., Gherzi R. Diverse roles of the nucleic acid-binding protein KHSRP in cell differentiation and disease. Wiley Interdiscip. Rev. RNA. 2016; 7:227–240. 26708421 10.1002/wrna.1327 PMC4770880

[41] Dong J. , Akcakanat A., Stivers D.N., Zhang J., Kim D., Meric-Bernstam F. RNA-binding specificity of Y-box protein 1. RNA Biol. 2009; 6:59–64. 19098458 10.4161/rna.6.1.7458 PMC4322766

[42] Kannan A. , Bhatia K., Branzei D., Gangwani L. Combined deficiency of Senataxin and DNA-PKcs causes DNA damage accumulation and neurodegeneration in spinal muscular atrophy. Nucleic Acids Res. 2018; 46:8326–8346. 30010942 10.1093/nar/gky641 PMC6144794

[43] Naumann M. , Pal A., Goswami A., Lojewski X., Japtok J., Vehlow A., Naujock M., Gunther R., Jin M., Stanslowsky N. et al. Impaired DNA damage response signaling by FUS-NLS mutations leads to neurodegeneration and FUS aggregate formation. Nat. Commun. 2018; 9:335. 29362359 10.1038/s41467-017-02299-1 PMC5780468

[44] Zhu L.S. , Wang D.Q., Cui K., Liu D., Zhu L.Q. Emerging perspectives on DNA double-strand breaks in neurodegenerative diseases. Curr. Neuropharmacol. 2019; 17:1146–1157. 31362659 10.2174/1570159X17666190726115623 PMC7057204

[45] Chen E.B. , Qin X., Peng K., Li Q., Tang C., Wei Y.C., Yu S., Gan L., Liu T.S. HnRNPR-CCNB1/CENPF axis contributes to gastric cancer proliferation and metastasis. Aging (Albany NY). 2019; 11:7473–7491. 31527303 10.18632/aging.102254 PMC6782008

[46] Huang J. , Li S.J., Chen X.H., Han Y., Xu P. hnRNP-R regulates the PMA-induced c-fos expression in retinal cells. Cell Mol. Biol. Lett. 2008; 13:303–311. 18197392 10.2478/s11658-008-0002-0 PMC6275800

[47] Reches A. , Nachmani D., Berhani O., Duev-Cohen A., Shreibman D., Ophir Y., Seliger B., Mandelboim O. HNRNPR regulates the expression of classical and nonclassical MHC class I proteins. J. Immunol. 2016; 196:4967–4976. 27194785 10.4049/jimmunol.1501550

[48] Fukuda A. , Nakadai T., Shimada M., Hisatake K. Heterogeneous nuclear ribonucleoprotein R enhances transcription from the naturally configured c-fos promoter in vitro. J. Biol. Chem. 2009; 284:23472–23480. 19581295 10.1074/jbc.M109.013656 PMC2749121

[49] Chabot B. , Blanchette M., Lapierre I., La Branche H. An intron element modulating 5′ splice site selection in the hnRNP A1 pre-mRNA interacts with hnRNP A1. Mol. Cell. Biol. 1997; 17:1776–1786. 9121425 10.1128/mcb.17.4.1776 PMC232024

[50] Solomon O. , Oren S., Safran M., Deshet-Unger N., Akiva P., Jacob-Hirsch J., Cesarkas K., Kabesa R., Amariglio N., Unger R. et al. Global regulation of alternative splicing by adenosine deaminase acting on RNA (ADAR). RNA. 2013; 19:591–604. 23474544 10.1261/rna.038042.112 PMC3677275

[51] Alemasova E.E. , Moor N.A., Naumenko K.N., Kutuzov M.M., Sukhanova M.V., Pestryakov P.E., Lavrik O.I. Y-box-binding protein 1 as a non-canonical factor of base excision repair. Biochim. Biophys. Acta (BBA)-Proteins Proteomics. 2016; 1864:1631–1640. 27544639 10.1016/j.bbapap.2016.08.012

[52] Alemasova E.E. , Naumenko K.N., Kurgina T.A., Anarbaev R.O., Lavrik O.I. The multifunctional protein YB-1 potentiates PARP1 activity and decreases the efficiency of PARP1 inhibitors. Oncotarget. 2018; 9:23349–23365. 29805738 10.18632/oncotarget.25158 PMC5955111

[53] Fomina E.E. , Pestryakov P.E., Maltseva E.A., Petruseva I.O., Kretov D.A., Ovchinnikov L.P., Lavrik O.I. Y-box binding protein 1 (YB-1) promotes detection of DNA bulky lesions by XPC-HR23B factor. Biochemistry (Mosc.). 2015; 80:219–227. 25756536 10.1134/S000629791502008X

[54] Gaudreault I. , Guay D., Lebel M. YB-1 promotes strand separation in vitro of duplex DNA containing either mispaired bases or cisplatin modifications, exhibits endonucleolytic activities and binds several DNA repair proteins. Nucleic Acids Res. 2004; 32:316–327. 14718551 10.1093/nar/gkh170 PMC373280

[55] Kim E.R. , Selyutina A.A., Buldakov I.A., Evdokimova V., Ovchinnikov L.P., Sorokin A.V. The proteolytic YB-1 fragment interacts with DNA repair machinery and enhances survival during DNA damaging stress. Cell Cycle. 2013; 12:3791–3803. 24107631 10.4161/cc.26670 PMC3905071

[56] Guay D. , Gaudreault I., Massip L., Lebel M. Formation of a nuclear complex containing the p53 tumor suppressor, YB-1, and the Werner syndrome gene product in cells treated with UV light. Int. J. Biochem. Cell Biol. 2006; 38:1300–1313. 16584908 10.1016/j.biocel.2006.01.008

[57] Dutertre M. , Lambert S., Carreira A., Amor-Guéret M., Vagner S. DNA damage: RNA-binding proteins protect from near and far. Trends Biochem. Sci. 2014; 39:141–149. 24534650 10.1016/j.tibs.2014.01.003

[58] Wang H. , Guo W., Mitra J., Hegde P.M., Vandoorne T., Eckelmann B.J., Mitra S., Tomkinson A.E., Van Den Bosch L., Hegde M.L Mutant FUS causes DNA ligation defects to inhibit oxidative damage repair in Amyotrophic Lateral Sclerosis. Nat. Commun. 2018; 9:3683. 30206235 10.1038/s41467-018-06111-6 PMC6134028

[59] Mitra J. , Guerrero E.N., Hegde P.M., Liachko N.F., Wang H., Vasquez V., Gao J., Pandey A., Taylor J.P., Kraemer B.C. et al. Motor neuron disease-associated loss of nuclear TDP-43 is linked to DNA double-strand break repair defects. Proc. Natl. Acad. Sci. U.S.A. 2019; 116:4696–4705. 30770445 10.1073/pnas.1818415116 PMC6410842

[60] Jangi M. , Fleet C., Cullen P., Gupta S.V., Mekhoubad S., Chiao E., Allaire N., Bennett C.F., Rigo F., Krainer A.R. et al. SMN deficiency in severe models of spinal muscular atrophy causes widespread intron retention and DNA damage. Proc. Natl. Acad. Sci. U.S.A. 2017; 114:E2347–E2356. 28270613 10.1073/pnas.1613181114 PMC5373344

[61] Kannan A. , Bhatia K., Branzei D., Gangwani L. Combined deficiency of Senataxin and DNA-PKcs causes DNA damage accumulation and neurodegeneration in spinal muscular atrophy. Nucleic Acids Res. 2018; 46:8326–8346. 30010942 10.1093/nar/gky641 PMC6144794

