# Supplementary material for: Loss of full-length hnRNP R isoform impairs DNA damage response in motoneurons by inhibiting Yb1 recruitment to chromatin
Source: Nucleic Acids Res. 2021 Nov 25;49(21):12284–305. doi: 10.1093/nar/gkab1120 (PMC8643683; doi:10.1093/nar/gkab1120)
Supplement: gkab1120_Supplemental_Files [file gkab1120_supplemental_files.zip › Supplemental Figures+Legends.pdf]

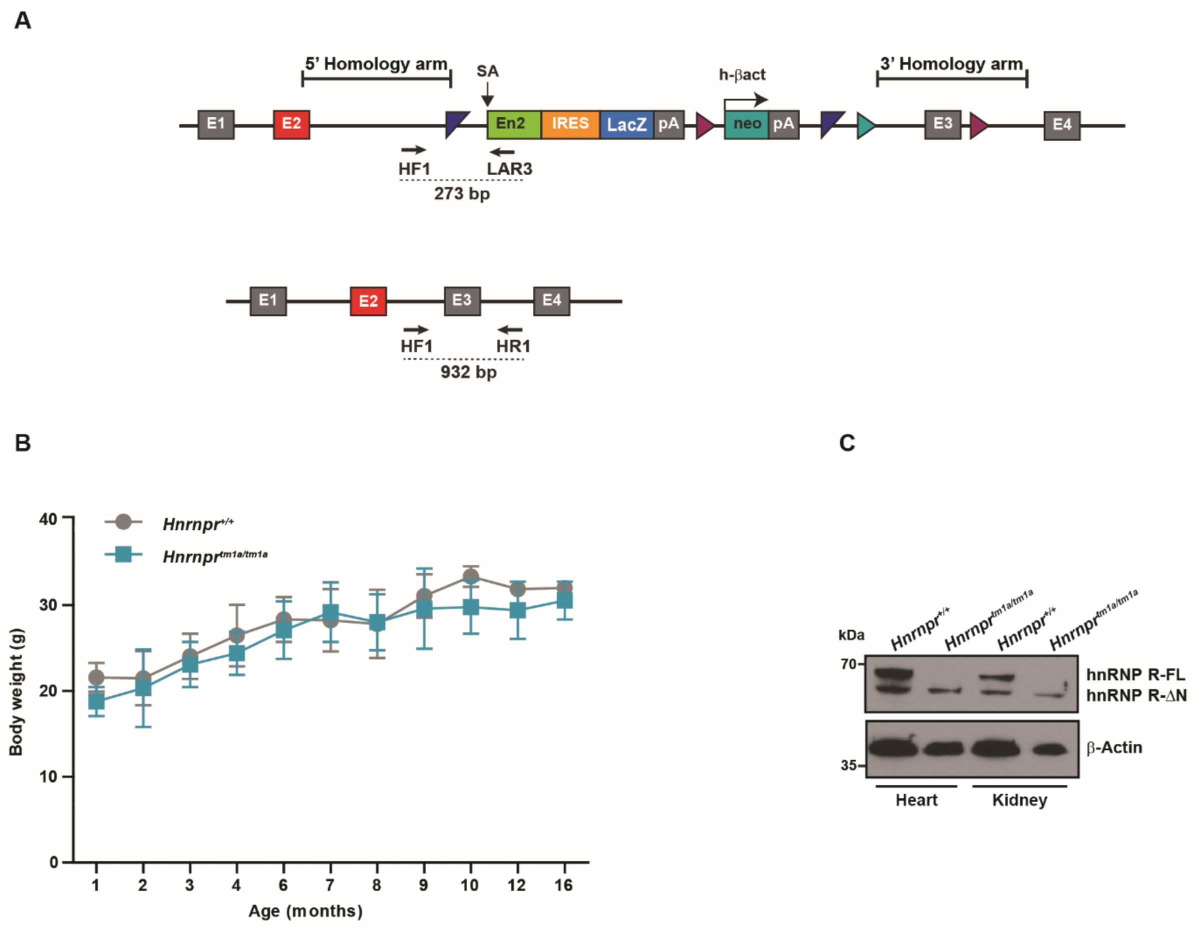

Supplemental Figure S1: Molecular and phenotypic characterization of *Hnrnpr*$^{tm1a/tm1a}$ mice.

(A) Long-range PCR was used to confirm the presence of the knockout allele (tm1a) using genomic DNA extracted from mouse tails. PCR primers used to genotype the target allele are shown beneath the diagram. Successful disruption of *Hnrnpr* gene was verified using a forward gene-specific primer located in the 5′ homology arm downstream of the cassette (HF1) and a vector specific reverse primer within the lacZ cassette (LAR3), generating a 273 bp product. To distinguish the wildtype allele, HF1 primer was paired with a reverse gene specific primer HR1 located in the 3′ homology arm in intron 3, yielding a PCR product of 932 bp. (B) Line graph plot of average body weight for *Hnrnpr*$^{+/+}$ and *Hnrnpr*$^{tm1a/tm1a}$ mice between the ages of 1 and 16 months. Data are mean ± SD (n=13 animals in *Hnrnpr*$^{+/+}$ and n=11 animals in *Hnrnpr*$^{tm1a/tm1a}$) (C) Western blot analysis of hnRNP R isoforms using a C-terminal specific antibody performed in tissues lysates prepared from P5 *Hnrnpr*$^{+/+}$ and *Hnrnpr*$^{tm1a/tm1a}$ mice. Equal protein content in the individual lanes was verified by probing with β-Actin antibody.

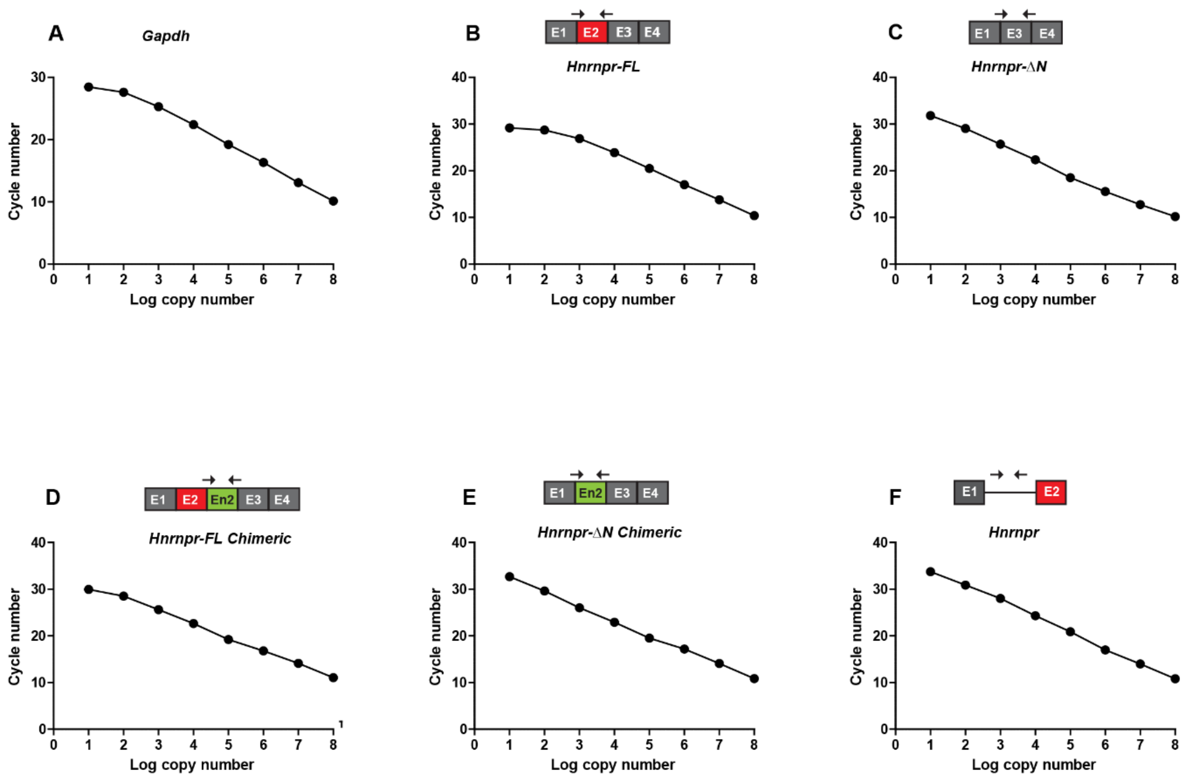

Supplemental Figure S2: Standard curves used for absolute quantification.

Standard curves for absolute quantification of *Gapdh* and *Hnrnpr* isoforms measured by qRT-PCR. Primers used are shown.

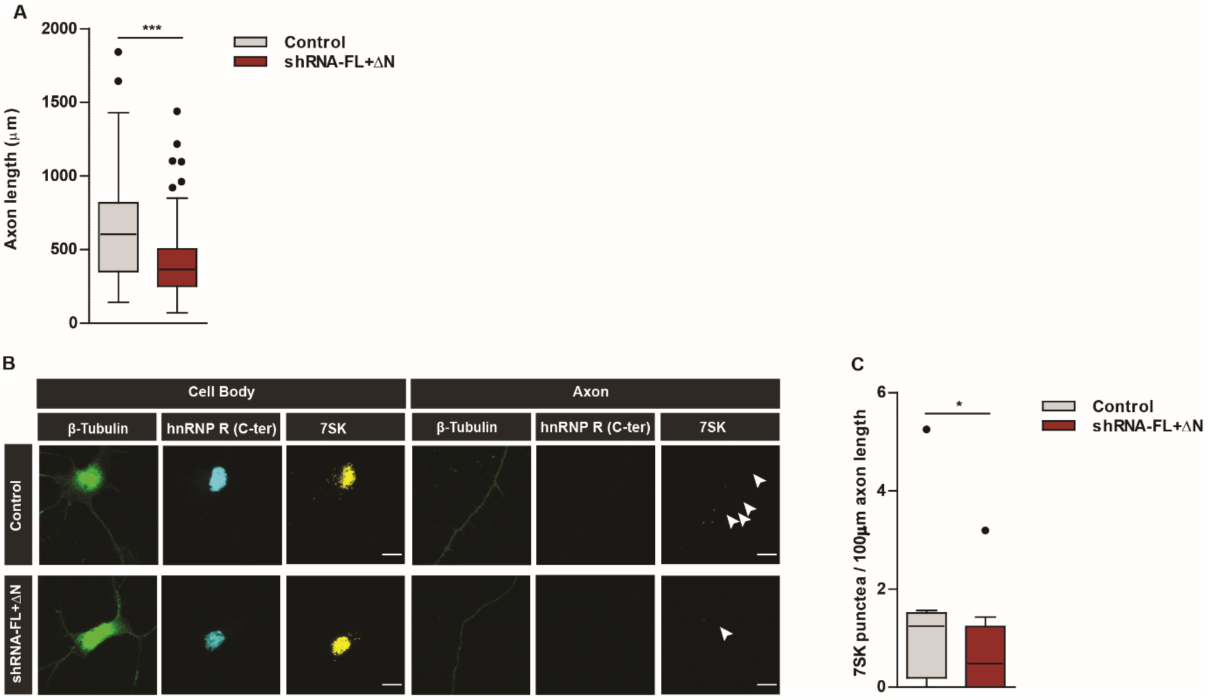

Supplemental Figure S3: knockdown of both hnRNP R isoforms results in shorter axons and disturbed 7SK translocation

(A) Box-and-whisker plots of the axon lengths of motoneurons transduced with control and shRNA targeting both hnRNP R isoforms (shRNA-FL+ΔN) cultured for 7 DIV. Data are mean ± SD (n=3 independent experiments; N=274 motoneurons for control and N=328 motoneurons for shRNA-FL+ΔN). Statistical analysis was performed using the Mann-Whitney test; ***p ≤ 0.001. (B) Representative images showing 7SK RNA labeling by *in situ* hybridization and immunostaining for hnRNP R in motoneurons transduced with control or shRNA-FL+ΔN lentivirus. Arrowheads show 7SK-positive punctae. Scale bars: 10 µm. (C) Box-and-whisker plots of the number of 7SK-positive punctae in axons cultured for 6 DIV. Data are mean ± SD (n=2 independent experiments; N=10 axons for control and N=10 axons for shRNA-FL+ΔN). Statistical analysis was performed using the Mann–Whitney test; *p ≤ 0.05.

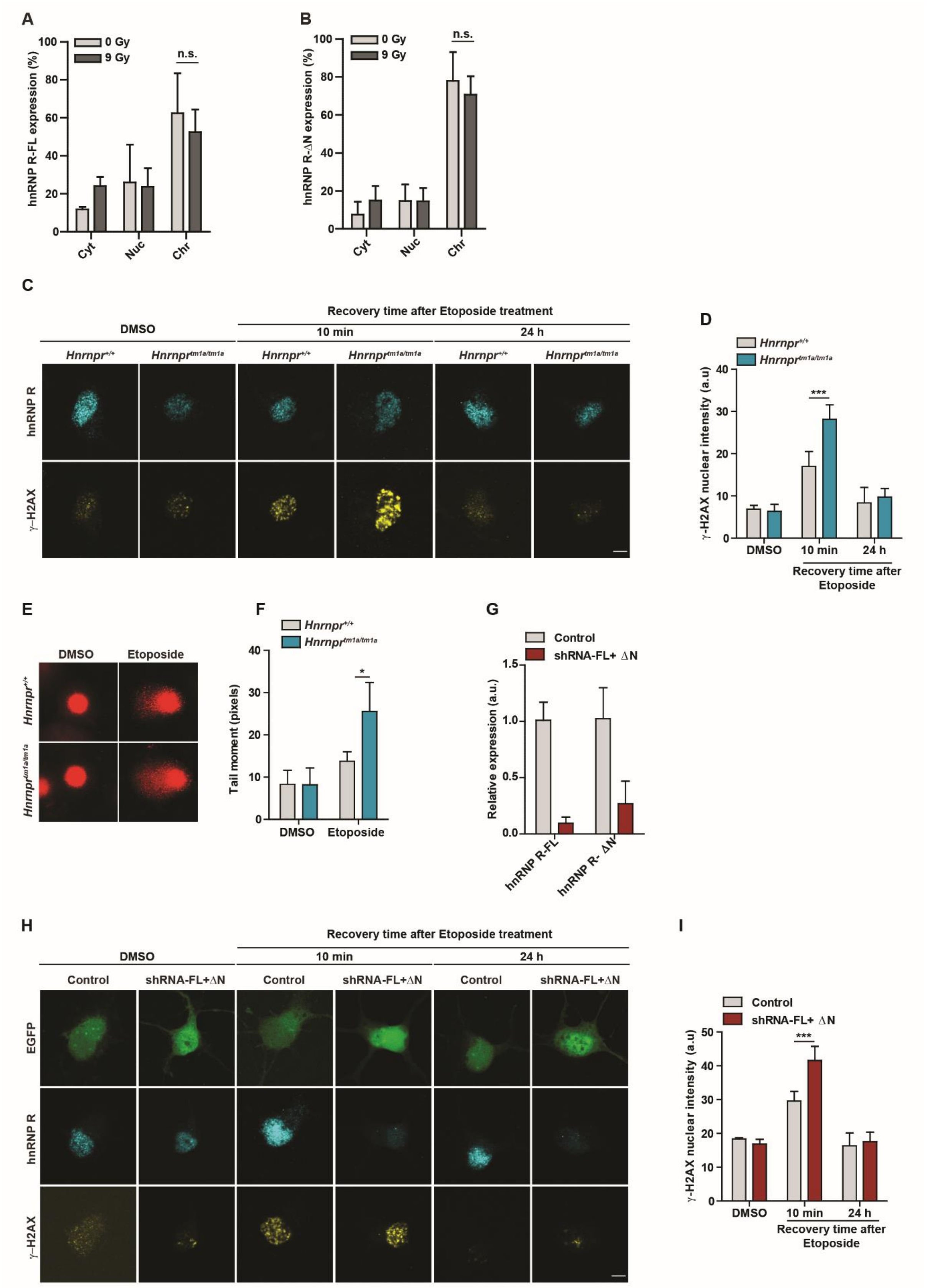

Supplemental Figure S4: Increased DNA damage and impaired DDR in *Hnrnpr*$^{tm1a//tm1a}$ motoneurons

(A,B) Quantification of Western blot data shown in (Figure 5A) for hnRNP R-FL (A) and hnRNP R-ΔN (B) isoforms. The expression of each isoform in indicated fraction is presented as the percentage of total. Data are mean ± SD (n=3 independent experiments). Statistical analysis was performed using one-way ANOVA followed by Tukey's Multiple Comparison Test; n.s., not significant. (C) Representative images of γ-H2AX immunofluorescence staining of *Hnrnpr*$^{+/+}$ and *Hnrnpr*$^{tm1a/tm1a}$ motoneurons treated with DMSO or etoposide (10 μM) for 14 h followed by indicated recovery times. Scale bars: 5 μm. (D) Quantification of nuclear γ-H2AX immunostaining in (C). Data are mean ± SD (n=3 independent experiments; N=27 nuclei for *Hnrnpr*$^{+/+}$ (DMSO), N=27 nuclei for *Hnrnpr*$^{tm1a/tm1a}$ (DMSO), N=27 nuclei for *Hnrnpr*$^{+/+}$ (Etoposide, 10 min and 24 h recovery time), N=27 nuclei for *Hnrnpr*$^{tm1a/tm1a}$ (Etoposide, 10 min and 24 h recovery time)). Statistical analysis was performed using two-way ANOVA followed by Bonferroni post-hoc test; ***p ≤ 0.001. (E) Representative images of alkaline comet assays performed on *Hnrnpr*$^{+/+}$ and *Hnrnpr*$^{tm1a/tm1a}$ motoneurons cultured for 6 DIV and treated with DMSO or etoposide (10 μM) for 14 h. (F) Quantification of comet mean tail moments. Data are mean ± SD (n=3 independent experiments; N=26 nuclei for *Hnrnpr*$^{+/+}$ (0 Gy), N=30 nuclei for *Hnrnpr*$^{+/+}$ (9 Gy), N=30 nuclei for *Hnrnpr*$^{tm1a/tm1a}$ (0 Gy) and N=30 nuclei for *Hnrnpr*$^{tm1a/tm1a}$ (9 Gy)). Statistical analysis was performed using two-way ANOVA followed by Bonferroni post-hoc test; *p ≤ 0.05. (G) Quantification of western blot data shown in (Figure 5H). Data are mean ± SD (n=3 independent experiments). (H) Representative images of γ-H2AX immunofluorescence staining of control and shRNA-FL+ΔN transduced motoneurons treated with DMSO or etoposide (10 μM) for 14 h followed by the indicated recovery time. EGFP was used as a marker to identify transduced cells. Scale bars: 5 μm. (I) Quantification of nuclear γ-H2AX immunostaining in (H). Data are mean ± SD (n=3 independent experiments; N=20 nuclei for control (DMSO), N=19 nuclei for control (Etoposide, 10 min recovery), N= 28 nuclei for control (Etoposide, 24 h recovery), N=18 nuclei for shRNA-FL+ΔN (DMSO), N=29 nuclei for shRNA-FL+ΔN (Etoposide, 10 min recovery), N=20 nuclei for shRNA-FL+ΔN (Etoposide, 24 h recovery)). Statistical analysis was performed using two-way ANOVA followed by Bonferroni post-hoc test; ***p ≤ 0.001.

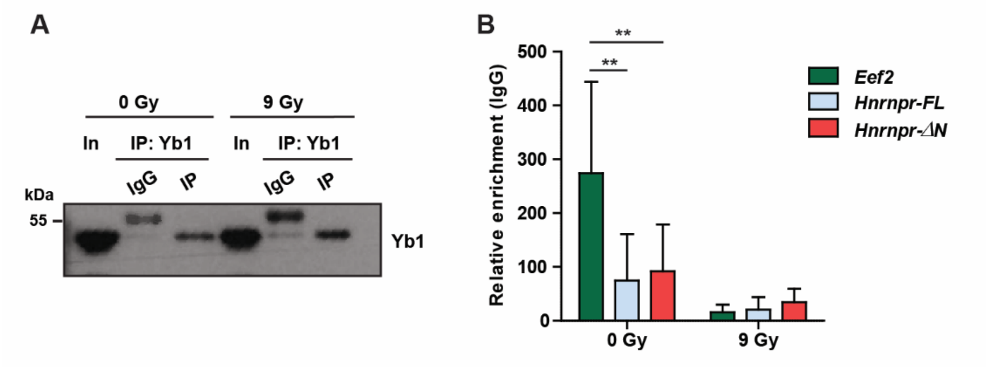

Supplemental Figure S5: Yb1 does not bind to the *Hnrnpr* transcripts

(A) Immunoprecipitation of Yb1 from lysates of control (0 Gy) or irradiated (9 Gy) NSC-34 cells. Proteins were analyzed by Western blot with antibodies against Yb1. In, input; IP, immunoprecipitate. Input represents 5% of the lysate used for immunoprecipitation. (B) qPCR analysis of *Eef2*, *Hnrnpr-FL* and *Hnrnpr-ΔN* transcripts co-immunoprecipitated by anti-Yb1 from control (0 Gy) or irradiated (9 Gy) NSC-34. Data are mean ± SD (n=4 independent experiments). Statistical analysis was performed using two-way ANOVA followed by Bonferroni post-hoc test; **p ≤ 0.01.

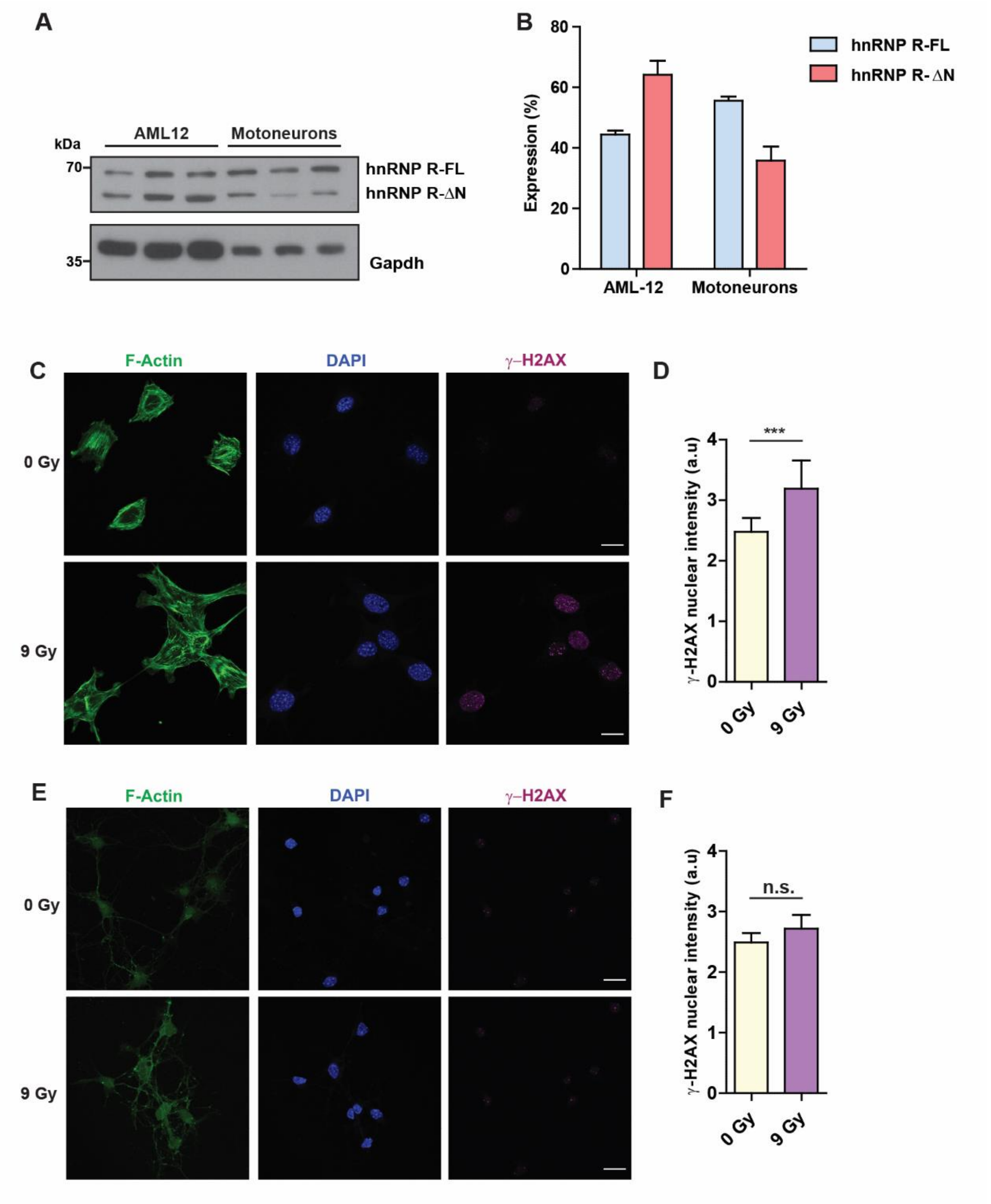

Supplemental Figure S6: Motoneurons have a high ability to repair DNA damage after irradiation

(A) Western blot analysis of hnRNP R isoforms using a C-terminal-specific antibody performed on AML12 liver cell and motoneuron lysates. Equal protein loading in individual lanes was verified by probing with an antibody against Gapdh. (B) Quantification of Western blot data in (A). The expression of each isoform is presented as the percentage of total levels. Data are mean ± SD (n=3 independent experiments). (C) Representative images of γ-H2AX immunofluorescence staining of AML12 cells under non-irradiated conditions (0 Gy) or after exposure to γ-irradiation (9 Gy) followed by 24 h recovery. Scale bars: 5 µm. (D) Quantification of nuclear γ-H2AX immunostaining in (C). Data are mean ± SD (n=2 independent experiments; N=35 nuclei for control (0 Gy), N=28 nuclei for irradiated (9 Gy)). Statistical analysis was performed using two-

way ANOVA followed by Bonferroni post-hoc test; ***p ≤ 0.001. (E) Representative images of γ-H2AX immunofluorescence staining of motoneurons under non-irradiated conditions (0 Gy) or after exposure to γ-irradiation (9 Gy) followed by 24 h recovery. Scale bars: 5 µm. (F) Quantification of nuclear γ-H2AX immunostaining in (E). Data are mean ± SD (n=3 independent experiments, N=15 nuclei for control (0 Gy), N=26 nuclei for irradiated (9 Gy)). Statistical analysis was performed using two-way ANOVA followed by Bonferroni post-hoc test. n.s., not significant.

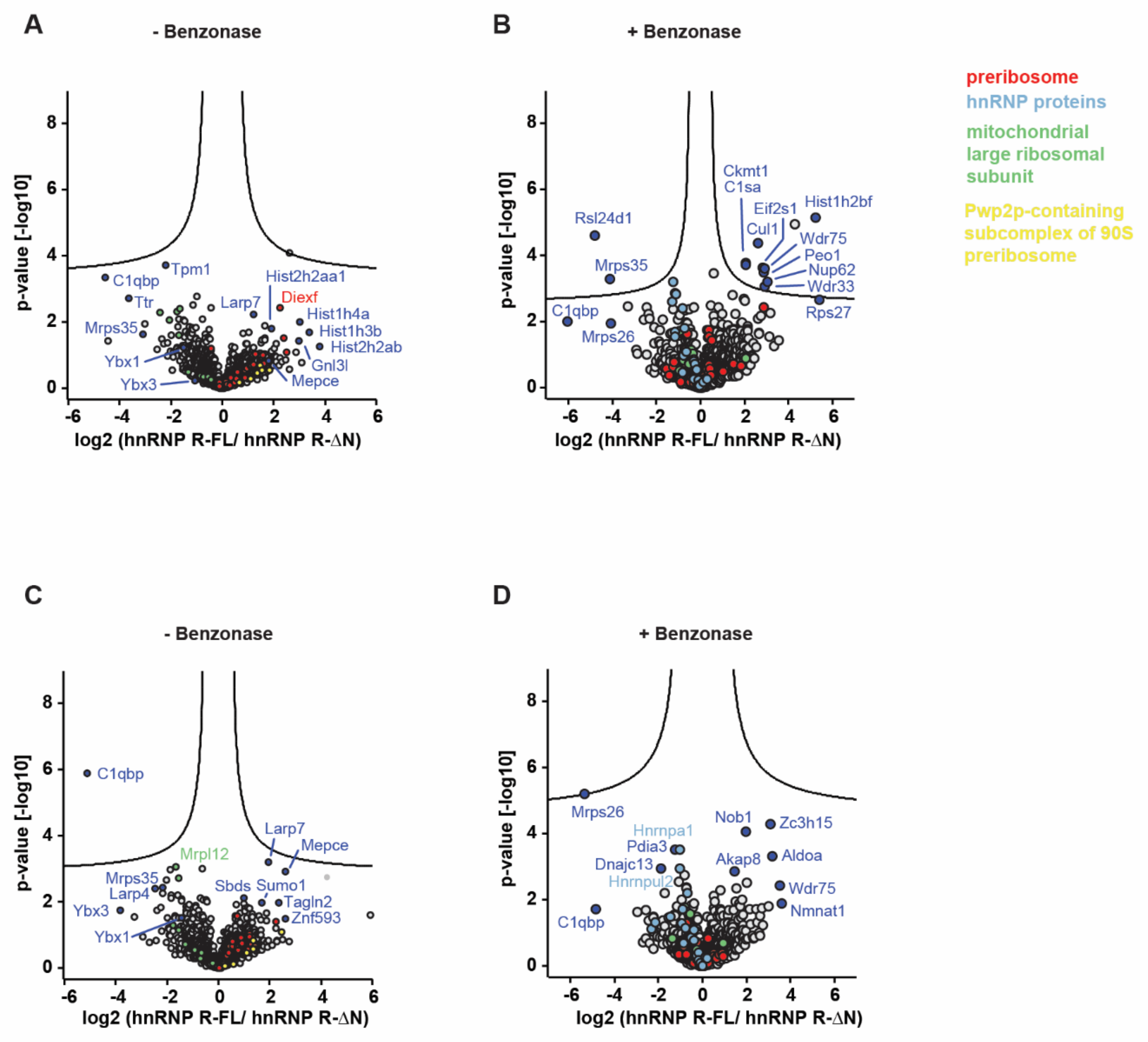

Supplemental Figure S7: Proteins enriched in hnRNP R-FL-HA vs. hnRNP R-ΔN-HA pulldown in HEK293TN cells and identified by LC-MS/MS.

Volcano plots showing proteins identified by mass spectrometry that were significantly enriched after immunoprecipitation with anti-HA antibody from HEK293TN transfected with plasmids for expression of hnRNP R-FL-HA or hnRNP R-ΔN-HA. Experiments were carried out in the absence (A and C) or presence (B and D) of Benzonase, under non-irradiated (0 Gy) (A and B) and irradiated (9 Gy) conditions (C and D). Data are shown as log-transformed fold changes in protein enrichment between hnRNP R-FL-HA and hnRNP R-ΔN-HA immunoprecipitations plotted against log-transformed p-values.
