# Supplementary material for: Loss of full-length hnRNP R isoform impairs DNA damage response in motoneurons by inhibiting Yb1 recruitment to chromatin
Source: Nucleic Acids Res. 2021 Nov 25;49(21):12284–305. doi: 10.1093/nar/gkab1120 (PMC8643683; doi:10.1093/nar/gkab1120)
Supplement: gkab1120_Supplemental_Files [file gkab1120_supplemental_files.zip › Supplemental tables description.docx]

**Description of Additional Supplementary information:**

Title: Supplemental Table S1

Description: List of proteins identified by mass spectrometry following hnRNP R immunoprecipitation from primary mouse motoneurons.

Title: Supplemental Table S2

Description: List of proteins identified by mass spectrometry following immunoprecipitation of HA-tagged hnRNP R isoforms or HA-EGFP control from irradiated or non-irradiated HEK293TN cells.
